# Supplementary material for: Optochemical profiling of NMDAR molecular diversity at synaptic and extrasynaptic sites
Source: EMBO J. 2025 Jul 8;44(16):4577–610. doi: 10.1038/s44318-025-00498-x (PMC12361563; doi:10.1038/s44318-025-00498-x)
Supplement: Supplementary file 1 — Appendix [file 44318_2025_498_MOESM1_ESM.pdf]

Appendix for:

Optochemical profiling of NMDAR molecular diversity at synaptic and extrasynaptic sites

Antoine Sicard, Meilin Tian, Zakaria Mostefai, Sophie Shi, Cécile Cardoso, Joseph Zamith, Isabelle McCort-Tranchepain, Cécile Charrier, Pierre Paoletti and Laetitia Mony

Table of Contents

Appendix Texts **2**
Appendix Text S1: NMR and mass-spectroscopy characterization of MASp 2
Appendix Spectra S1: NMR characterization of MASp 3
Appendix Spectra S2: Mass spectrometry characterization of MASp 7
Appendix Text S2: Photoswitching kinetics in mammalian cells 8
Appendix Text S3: Agonist-dependence of UV-induced potentiation 8

Appendix Figures **10**
Appendix Figure S1: Up to 3-fold potentiation of GluN2B diheteromer currents in HEK cells 10
Appendix Figure S2: Strong photomodulation of GluN2B*-R187C diheteromers in cultured cortical neurons 12
Appendix Figure S3: (related to Fig. 3) 13
Appendix Figure S4: Stability of Opto2B photoenhancement depends on glutamate concentration 15
Appendix Figure S5: Design strategy of the Opto2B mouse line: neutralizing the endogenous cysteine C395 is not necessary to obtain strong photomodulation in native preparations 17
Appendix Figure S6: Chemical structures of the four diastereoisomeric products of the reaction between MASp and L-Cysteine 19

Appendix Tables **20**
Appendix Table S1: Summary of in vitro photomodulation data 20
Appendix Table S2: Summary of several pharmacological parameters of 2B*-Q180C and 2B*-R187C mutants unlabeled (- MASp) and labeled (+ MASp) with MASp under different illumination conditions 21
Appendix Table S3: Average current values for MASp-labeled NMDAR constructs expressed in Xenopus oocytes 21
Appendix Table S4: Average current values for MASp-labeled NMDAR constructs expressed in HEK cells 21
Appendix Table S5: Average current values for MASp-labeled, electroporated and non-electroporated, cultured cortical neurons 22
Appendix Table S6: Summary of ex vivo photomodulation data on cortical neurons 22
Appendix Table S7: Summary of ex vivo photomodulation data in hippocampal CA1 pyramidal cells 23
Appendix Table S8: Average NMDA-EPSC values in hippocampal CA1 pyramidal neurons 23

Appendix References **23**

Appendix Texts

Appendix Text S1: NMR and mass-spectroscopy characterization of MASp

NMR spectra ^{1}H (500.16 MHz) and ^{13}C (125.78 MHz) for *trans* and *cis* MASp were recorded on a 500 Bruker spectrometer equipped with a sensitivity-optimized measurement head (cryoprobe). Chemical shifts (δ, ppm) are given with reference DMSO-*d*6 for ^{1}H and ^{13}C NMR, respectively: 2.50, 39.51. Signal multiplicity is described as follows: s (singlet), d (doublet), t (triplet), and m (multiplet). Broad signals are described as br. Coupling constants (J) are given in hertz. Molecule numbering is only related to atom assignment, which was established on the basis of ^{13}C using ^{1}H decoupled spectra as well as correlation spectroscopy, heteronuclear single quantum coherence, and heteronuclear multiple bond coherence.

Mass spectra were recorded on an Orbitrap Exactive (Thermoscientifc) mass spectrometer with positive (ESI+) electrospray ionization (ionization tension, 3.2 kV; ion transfer tube temperature, 275 °C). HPLC-MS analysis was performed on an Orbitrap Exactive Instrument as described above, equipped for HPLC (Nexera X2, Shimadzu) with a Phenomenex Kinetex C18 column (50 mm × 2.1 mm, 2.6 μm). MASp was eluted with the following gradient using solvent A (H_2O/HCO_2H: 100/0.1) and solvent B ($MeCN/HCO_2H$: 100/0.1), flow rate: 0.2 mL/min: 2% B linear increase to 100% B for 15 min, 100% B from 15 to 20 min, and linear decrease to 2% B from 20 to 20.01 min.

^{1}H NMR (500 MHz, DMSO-*d*$_6$) δ: 9.16 (brs, 2H, NH_2^+), 8.93 (brs, 4H, $2NH_2^+$), 8.02 (brs, 3H, NH_3^+), 7.87 (d, $J_{H\text{-}10,H\text{-}11}$ = 9.0 Hz, 1H, H-10), 7.82 (d, $J_{H\text{-}7,H\text{-}6}$ = 9.0 Hz, 1H, H-7), 7.17 (d, $J_{H\text{-}11,H\text{-}10}$ = 9.0 Hz, 1H, H-11), 7.07 (d, $J_{H\text{-}6,H\text{-}7}$ = 9.0 Hz, 1H, H-6), 7.06 (s, 1H, H-2), 4.36 (t, $J_{H\text{-}13,H\text{-}14}$ = 5.0 Hz, 2H, H-13), 4.22 (t, $J_{H\text{-}4,H\text{-}3}$ = 5.5 Hz, 2H, H-4), 3.83 (t, $J_{H\text{-}3,H\text{-}4}$ = 5.5 Hz, 2H, H-3), 3.42 (t, $J_{H\text{-}14,H\text{-}13}$ = 5.0 Hz, 2H, H-14), 3.12 (t, $J_{H\text{-}24,H\text{-}23}$ = 7.5 Hz, 2H, H-24), 3.02 (t, $J_{H\text{-}22,H\text{-}23}$ = 7.5 Hz, 2H, H-22), 2.99 (t, $J_{H\text{-}15,H\text{-}16}$ = 7.5 Hz, 2H, H-15), 2.93 (m, 4H, H-18, H-21), 2.89 (t, $J_{H\text{-}17,H\text{-}16}$ = 7.5 Hz, 2H, H-17), 2.02 (tt, $J_{H\text{-}23,H\text{-}22}$ = $J_{H\text{-}23,H\text{-}24}$ = 7.5 Hz, 2H, H-23), 1.90 (tt, $J_{H\text{-}16,H\text{-}15}$ = $J_{H\text{-}16,H\text{-}17}$ = 7.5 Hz, 2H, H-16), 1.64 (m, 4H, H-19, H-20).

^{13}C NMR (126 MHz, DMSO-*d*$_6$) δ: 170.9 (C-1), 160.3 (C-5), 159.9 (C-12), 158.7 (q, $^{3}J_{C,F}$ = 31.5 Hz, CO_2^-), 146.6 (C-9), 146.3 (C-8), 134.7 (C-2), 124.2 (C-7), 124.1 (C-10), 117.1 (q, $^{1}J_{C,F}$ = 299.5 Hz, C-F), 115.2 (C-11), 115.1 (C-6), 65.0 (C-4), 63.8 (C-13), 46.1 (C-18, C-21), 46.0 (C-14), 44.3 (C-24), 43.9, 43.8 (C-22, C-15), 36.6 (C-3), 36.2 (C-17), 23.8 (C-16), 22.7 (C-19, C-20), 22.4 (C-23).

HRMS (ESI+) m/z: calcd for $[C_{30}H_{43}N_7O_4 + H]^+$, 566.3449; found, 566.3436 (−2.2954 ppm); calcd for $[C_{30}H_{43}N_7O_4 + 2H]^{2+}$, 283.6761; found, 283.6754 (−2.4676 ppm); HPLC-MS (ESI) m/z (λ = 235 nm): RT = 6.76 min; 283.6755 $[M + 2H]^{2+}$ (−2.2637 ppm).

Appendix Spectra S1: NMR characterization of MASp

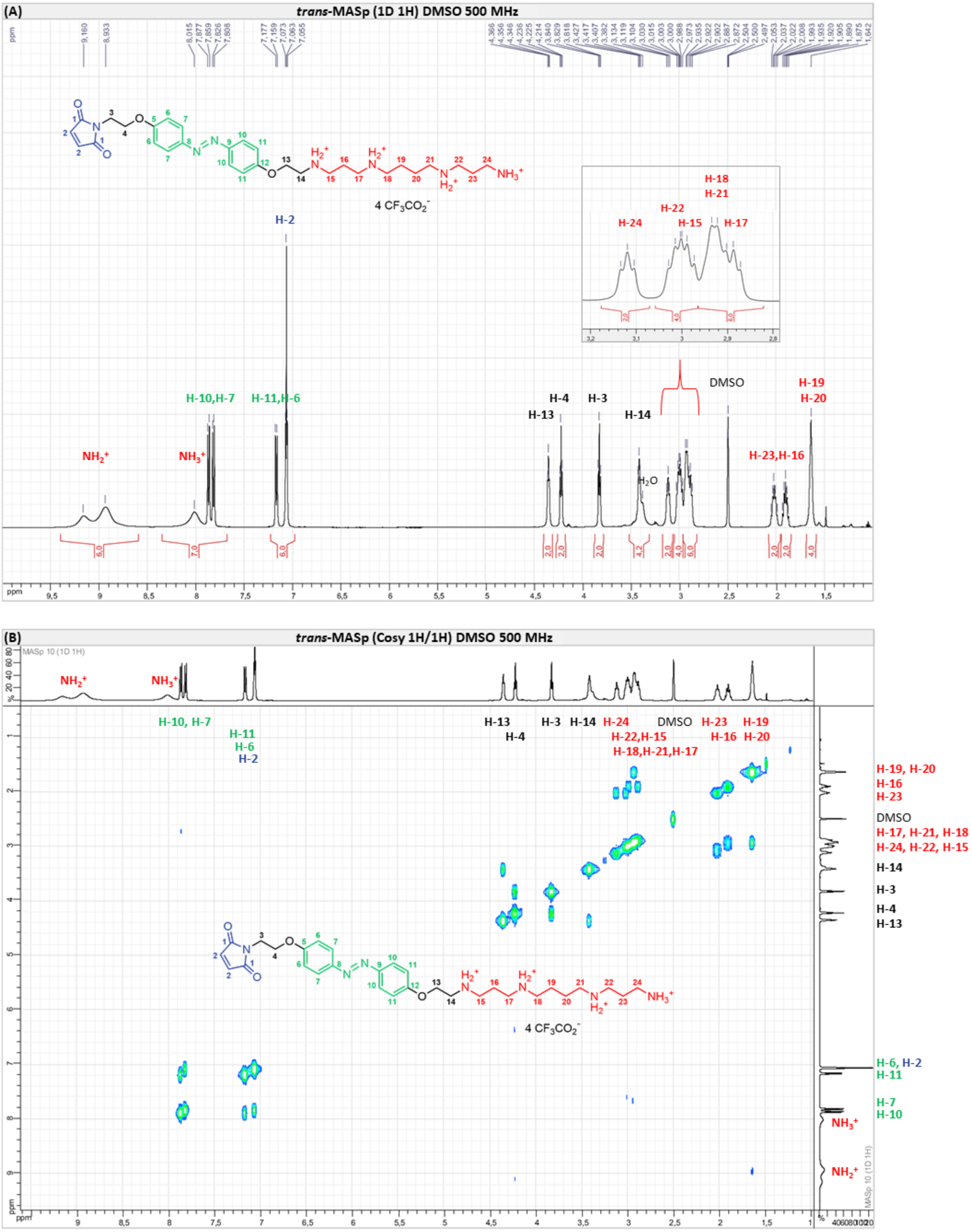

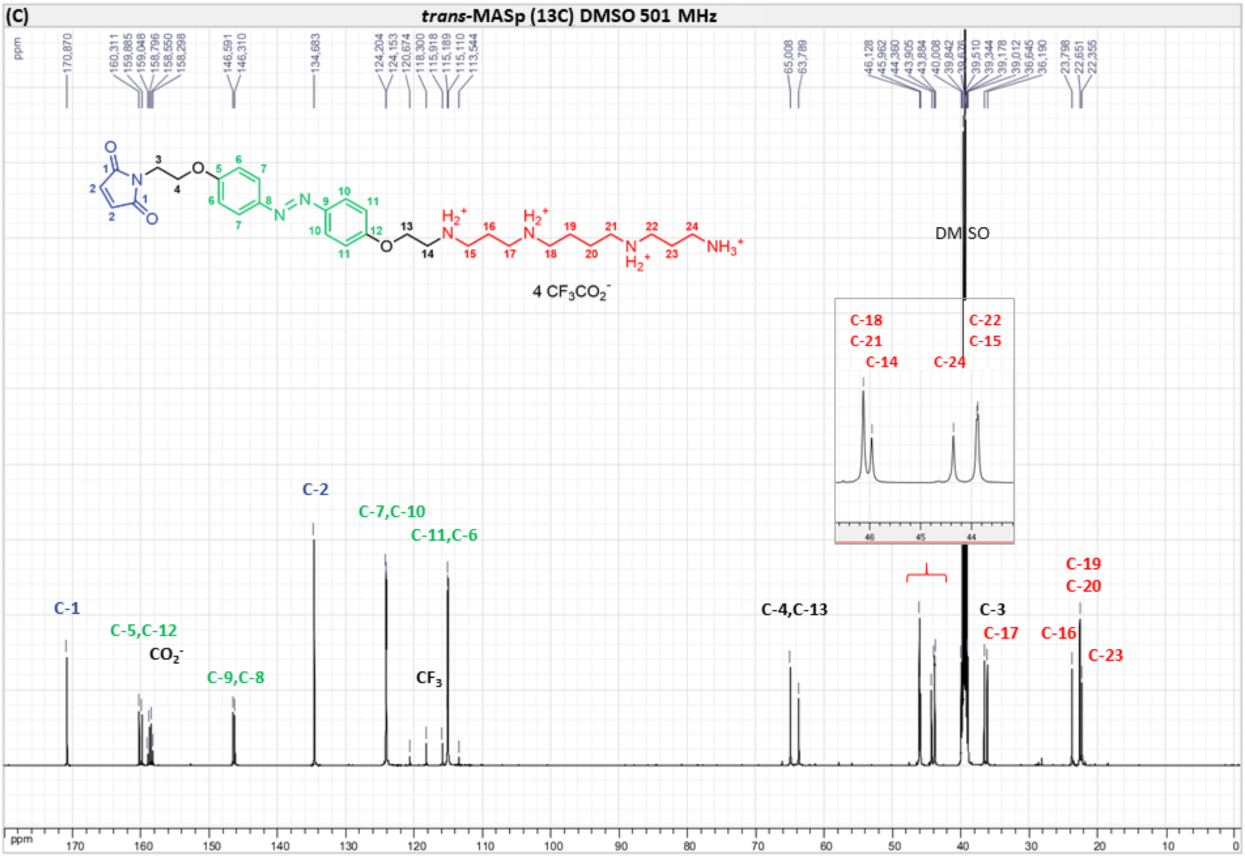
(C)
trans-MASp (13C) DMSO 501 MHz
ppm
170,870
160,311
159,885
159,048
158,796
158,550
158,298
146,591
146,310
134,683
124,204
124,153
120,674
118,300
115,918
115,189
115,110
113,544
65,008
63,789
46,128
45,962
44,360
43,905
43,884
40,008
39,842
39,676
39,510
39,344
39,178
39,012
36,645
36,190
23,798
22,651
22,355
4 CF3CO2-
DMSO
C-18
C-21
C-14
C-24
C-22
C-15
C-2
C-7,C-10
C-11,C-6
C-1
C-5,C-12
CO2-
C-9,C-8
CF3
C-4,C-13
C-3
C-17
C-16
C-19
C-20
C-23
170
160
150
140
130
120
110
100
90
80
70
60
50
40
30
20
10
0

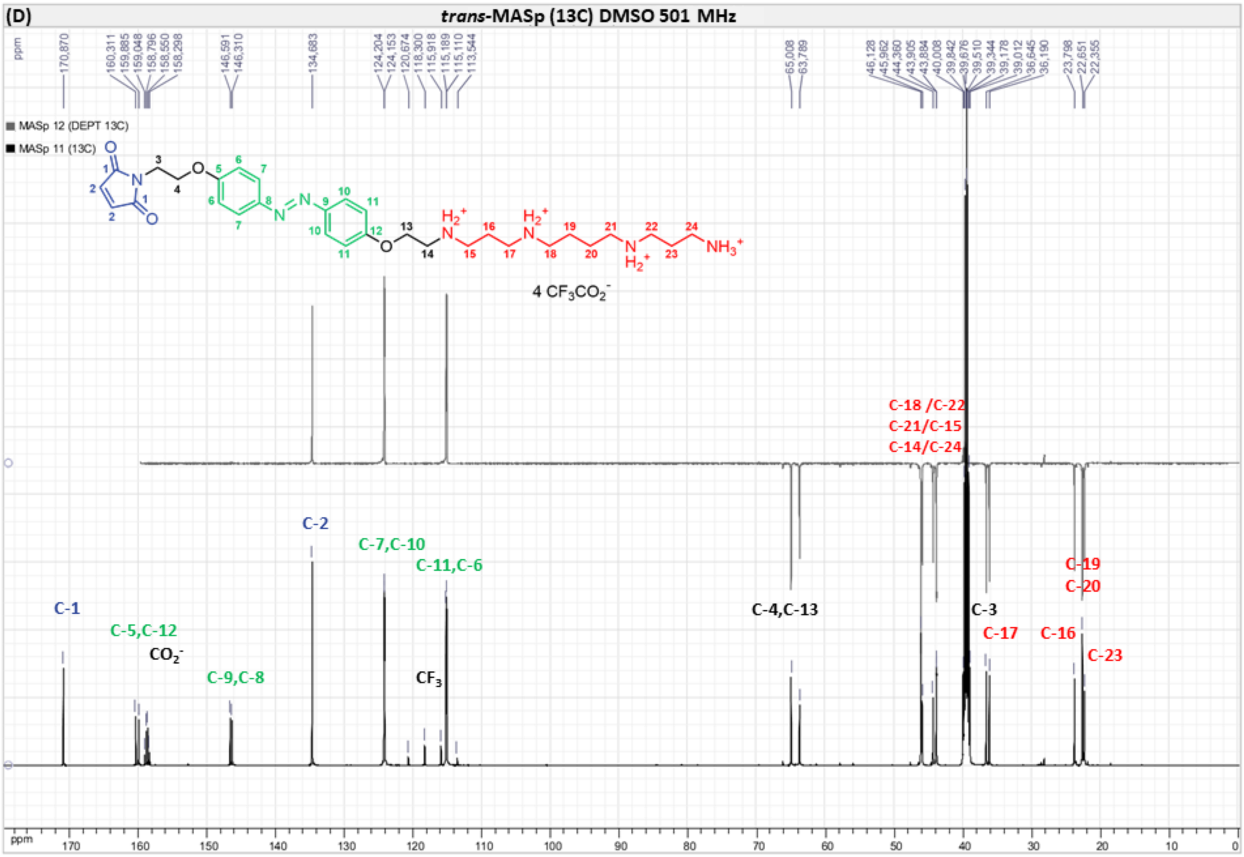
(D)
trans-MASp (13C) DMSO 501 MHz
ppm
170,870
160,311
159,885
159,048
158,796
158,550
158,298
146,591
146,310
134,683
124,204
124,153
120,674
118,300
115,918
115,189
115,110
113,544
65,008
63,789
46,128
45,962
44,360
43,905
43,884
40,008
39,842
39,676
39,510
39,344
39,178
39,012
36,645
36,190
23,798
22,651
22,355
MASp 12 (DEPT 13C)
MASp 11 (13C)
4 CF3CO2-
C-18 /C-22
C-21/C-15
C-14/C-24
C-2
C-7,C-10
C-11,C-6
C-1
C-5,C-12
CO2-
C-9,C-8
CF3
C-4,C-13
C-3
C-17
C-16
C-19
C-20
C-23
170
160
150
140
130
120
110
100
90
80
70
60
50
40
30
20
10
0

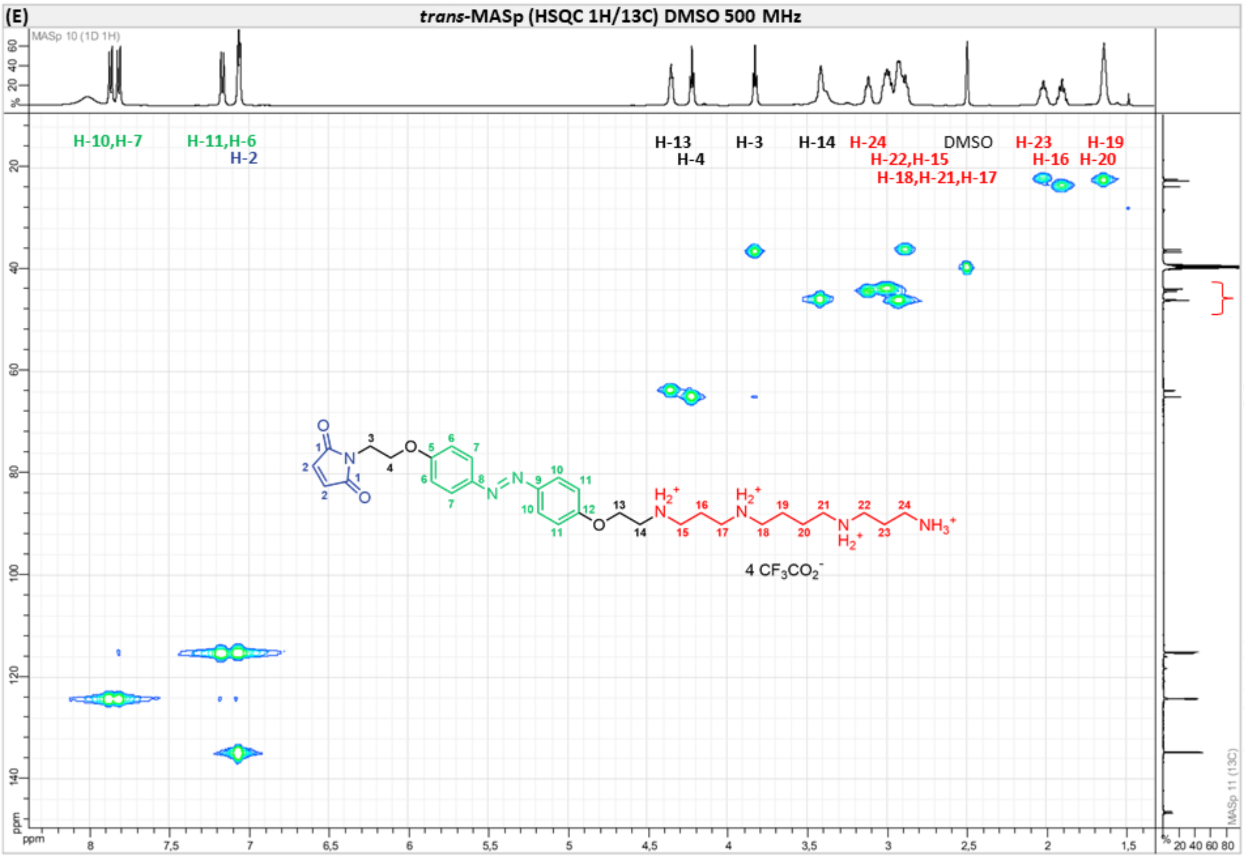
(E)
trans-MASp (HSQC 1H/13C) DMSO 500 MHz
H-10,H-7
H-11,H-6
H-2
H-13
H-4
H-3
H-14
H-24
H-22,H-15
H-18,H-21,H-17
DMSO
H-23
H-16
H-19
H-20
C-23
C-19+C-20
C-16
C-17
C-3
C-22,C-15
C-24
C-13
C-18+C-21
C-13
C-4
C-6
C-11
C-10
C-7
C-2
4 CF3CO2-
ppm

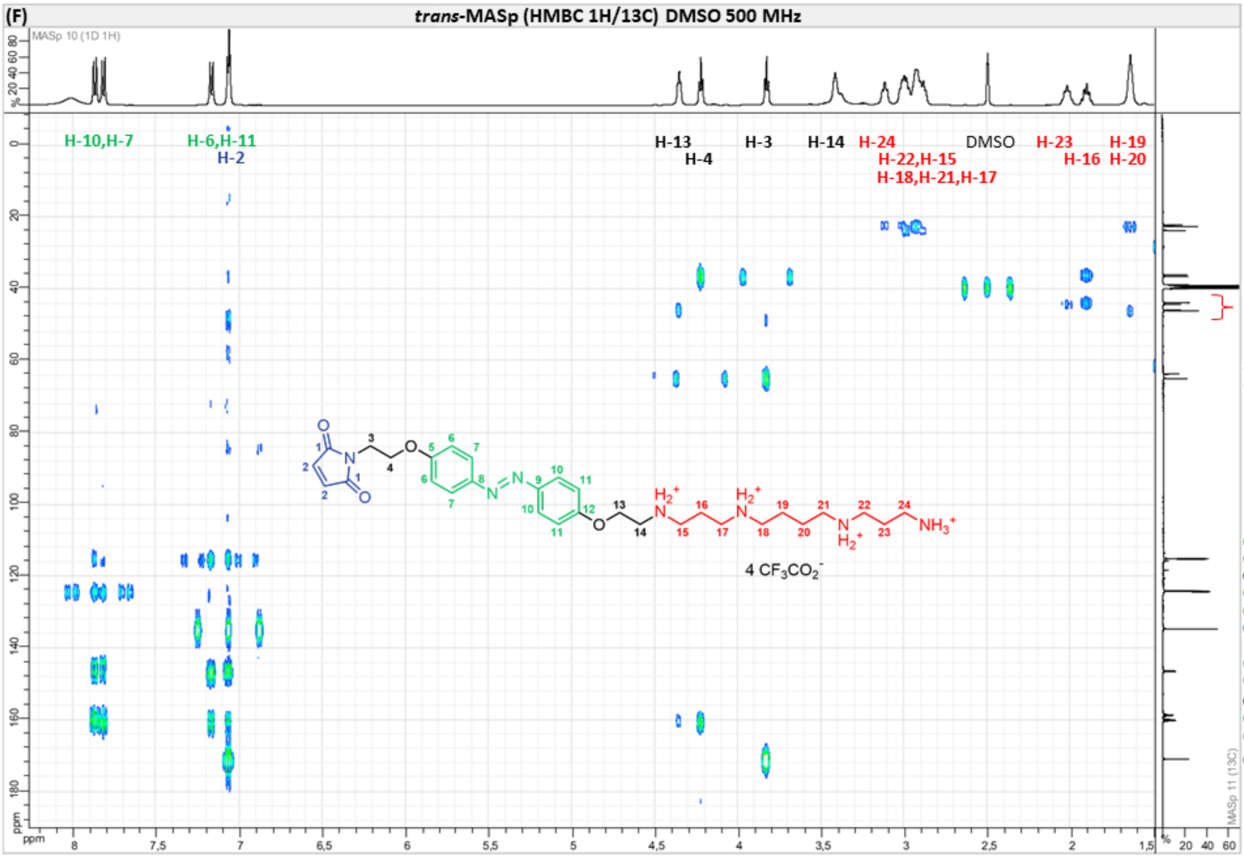
(F)
trans-MASp (HMBC 1H/13C) DMSO 500 MHz
H-10,H-7
H-6,H-11
H-2
H-13
H-4
H-3
H-14
H-24
H-22,H-15
H-18,H-21,H-17
DMSO
H-23
H-16
H-19
H-20
C-23
C-19+C-20
C-16
C-17
C-3
C-22,C-15
C-24
C-13
C-18+C-21
C-13
C-4
C-6
C-11
CF3
C-10
C-7
C-2
C-8
C-9
CO2-
C-12
C-5
C-1
4 CF3CO2-
ppm

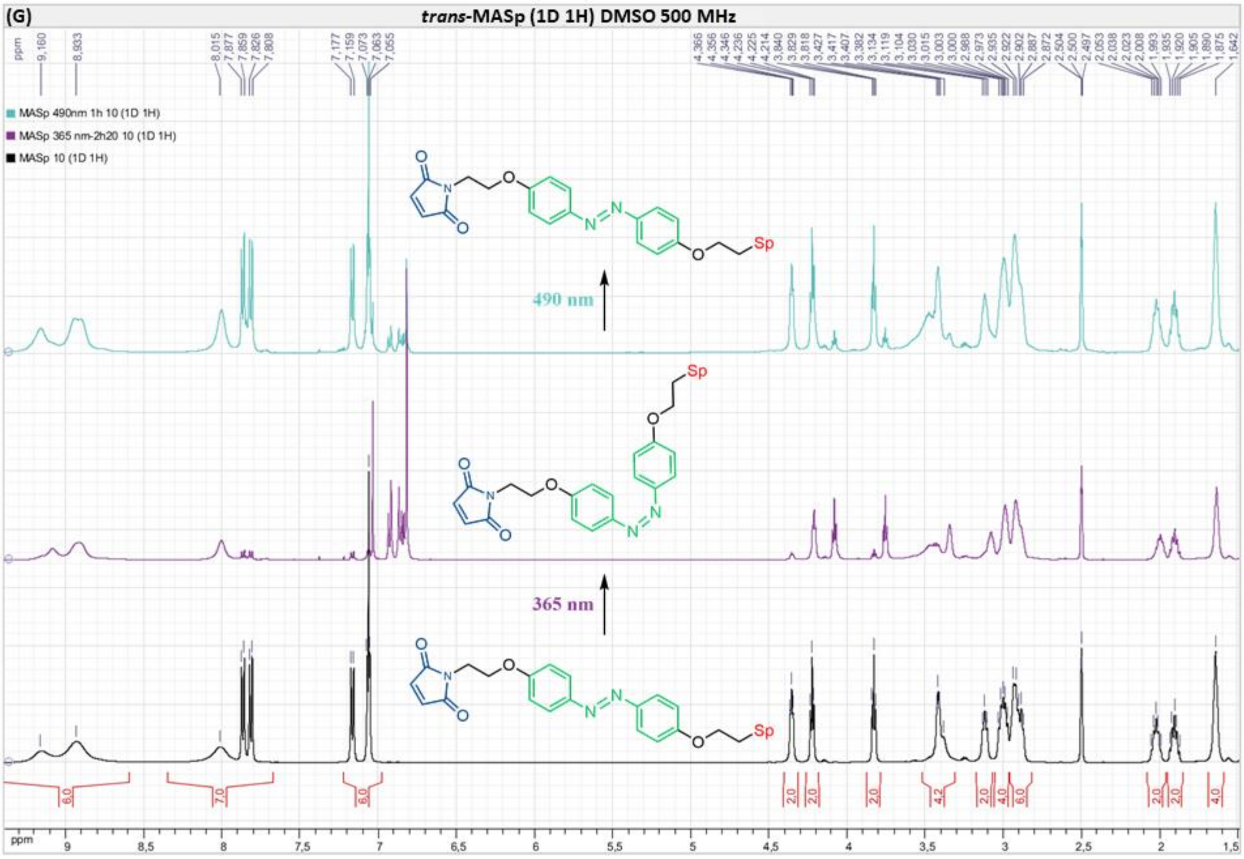

Lower panel: ^{1}H NMR spectrum of MASp in the dark (100% *trans*). Middle panel: ^{1}H NMR spectrum of MASp 365 nm PSS. Upper panel: ^{1}H NMR spectrum of MASp 490 nm PSS.

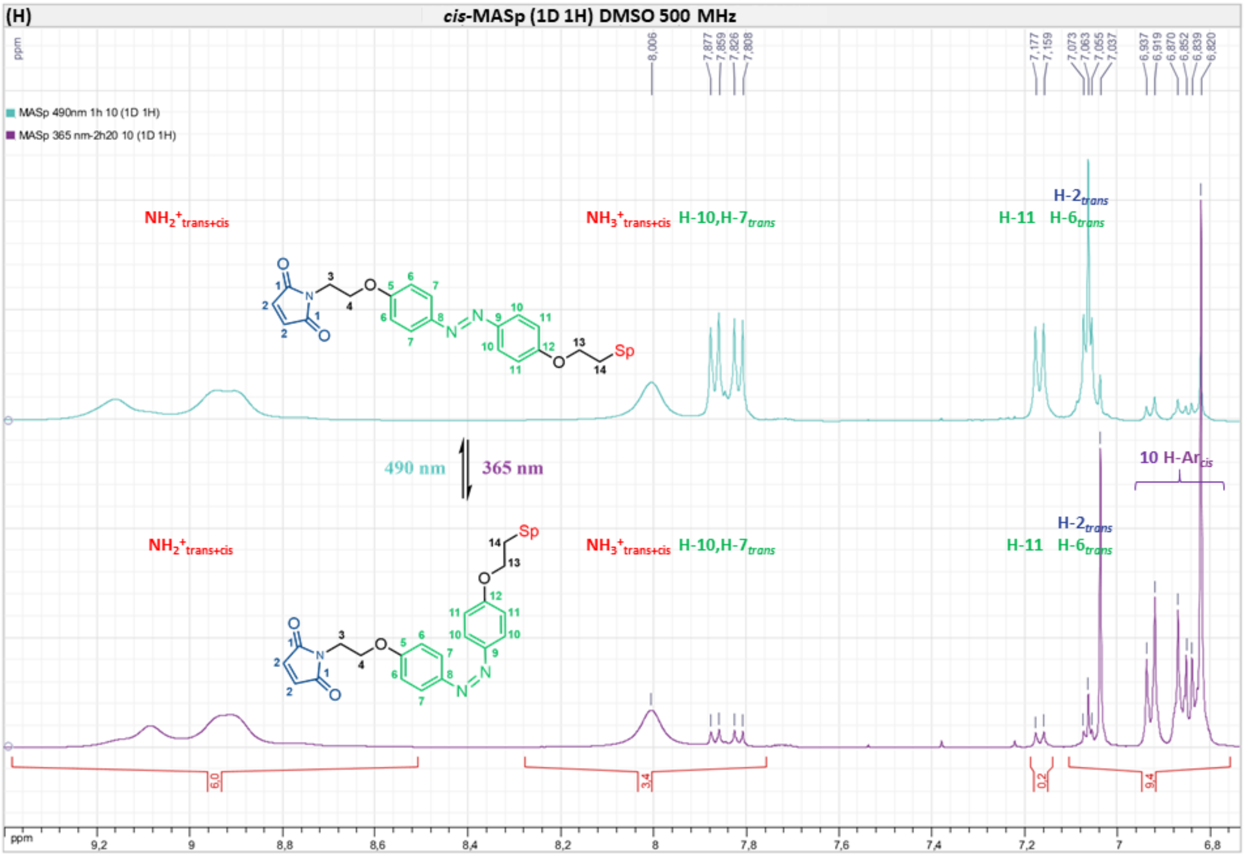

Percentage of *cis* and *trans* isomers PSSs was calculated from the integration of the peaks at 8.20−7.80 ppm and 7.16 ppm. Lower panel: 90% *cis* and 10% *trans*. Upper panel: 91% *trans* and 9% *cis*.

Appendix Spectra S2: Mass spectrometry characterization of MASp

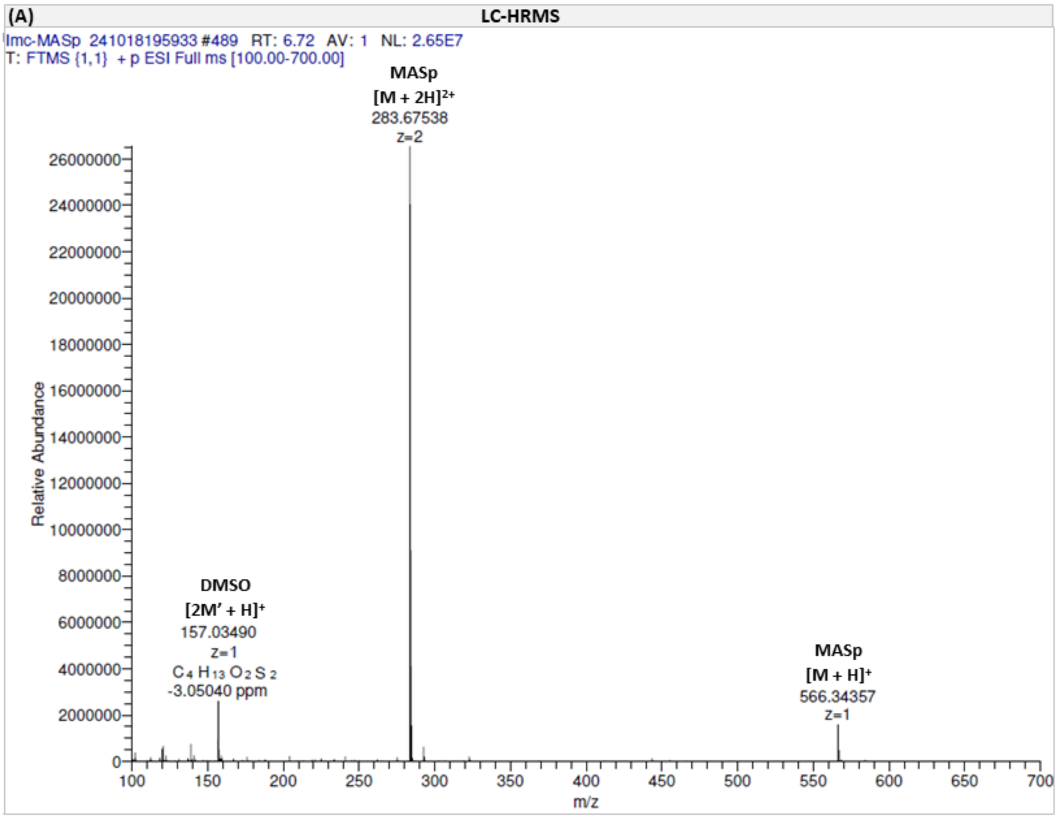

Full-MS spectrum at 6.72 min retention time showing essentially z = 2.

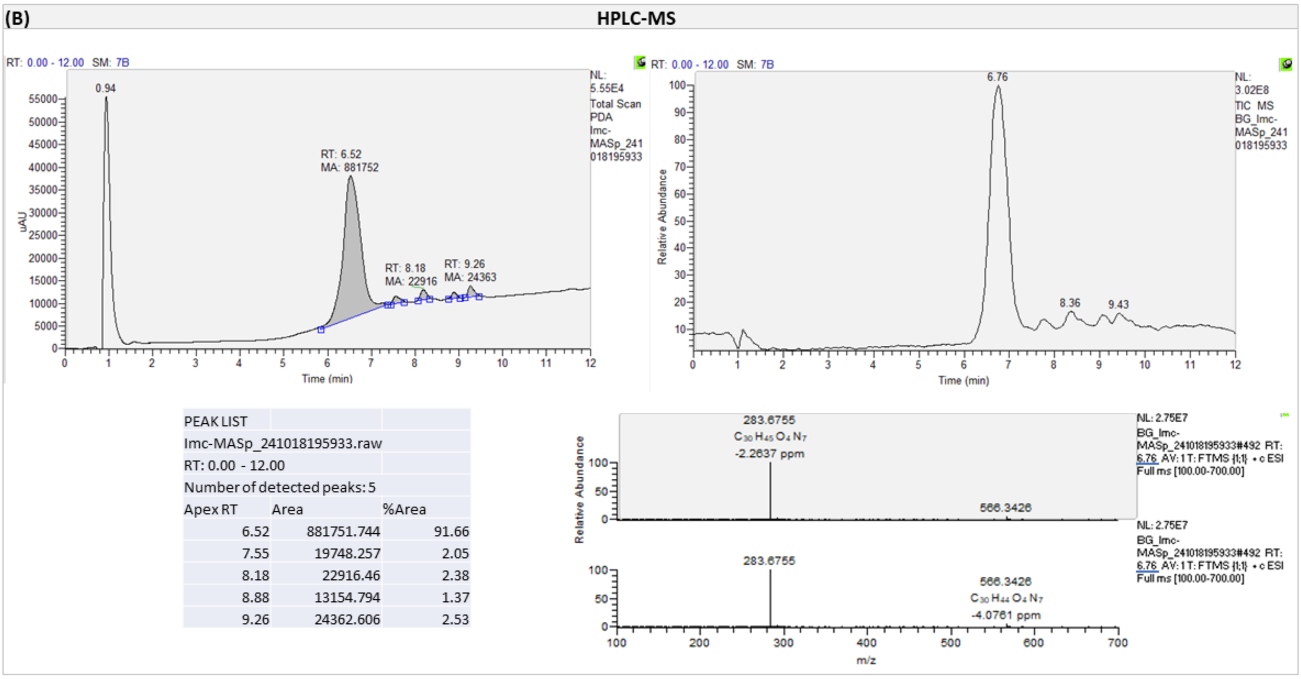

PEAK LIST		
Imc-MASp_241018195933.raw		
RT: 0.00 - 12.00		
Number of detected peaks: 5		
Apex RT	Area	%Area
6.52	881751.744	91.66
7.55	19748.257	2.05
8.18	22916.46	2.38
8.88	13154.794	1.37
9.26	24362.606	2.53

Left panel: UV detection chromatogram (PDA). Right panel: Mass spectrum peaks (TIC).

Appendix Text S2 (related to Appendix Fig. S1): Photoswitching kinetics in mammalian cells

At the maximal light power in our experimental conditions (~10 mW for 365 nm and 27 mW for 525 nm), receptor activity could be potentiated by UV light with a time constant of ~60 ms (MASp *trans*-to-*cis* transition) and reverted back to its basal state by 525 nm light with a much slower time constant of ~450 ms (MASp *cis*-to-*trans* transition; Appendix Fig. S1E,F). Strikingly, we observed that using blue light (460 nm) instead of green light (525 nm) to induce MASp *trans*-to-*cis* transition allowed a much faster (30-fold) reversal of receptor potentiation (time constant of ~13 ms; Appendix Fig. S1E,F), indicating that the slow transition under 525 nm light does not reflect slow kinetics of receptor conformational changes. Current off-rates with 525 nm light were systematically slower than current off-rates with 460 nm light regardless of the light power used (Appendix Fig. S1G). Hence, the wavelength-dependence of receptor off-rate kinetics is likely due to the much smaller absorbance of *cis*-MASp at 525 nm compared to 460 nm (*cis*-MASp absorptivity ε ~ 1900 $L.mol^{-1}.cm^{-1}$ at 460 nm and ε ~ 200 $L.mol^{-1}.cm^{-1}$ at 525 nm; calculated from the 365 nm PSS spectrum of Fig. EV1A), which results in a smaller probability of the molecule to undergo the *cis*-to-*trans* transition at 525 nm relative to 460 nm. Return to basal activity was however less complete under 460 nm light compared to 525 nm light (Appendix Fig. S1E,F), consistently with the lower proportion of the *trans* isomer in the 460 nm PSS compared to the 525 nm PSS (as assessed by UV-visible spectroscopy; Fig. EV1A). MASp thus allows very fast modification of GluN1/GluN2B diheteromer activity with on and off kinetics that can be below 100 ms. Depending on the requirements of the biological experiment, reversal of receptor potentiation can be performed either with blue light to achieve very fast reversal, or with green light to achieve more complete reversal to the basal state at the cost of a slower recovery. In the paper we will favor the second option and use 365 nm and 525-530 nm as the two photoswitching wavelengths.

Appendix Text S3 (related to Appendix Fig. S4). Agonist-dependence of UV-induced potentiation

To quantify the amount of photomodulation of tonic currents by UV light, we first assessed the glutamate-dependence of the photomodulation ratio when measured at the peak or steady-state UV-potentiation. For this we turned to Xenopus oocytes in order to obtain expression of a pure population of GluN2B*-R187C diheteromers. Similarly to neurons, we observed on this system sustained UV potentiation at saturating glutamate concentrations (100 µM) and a transient potentiation at glutamate concentrations lower than the receptor EC_{50} (0.03 and 0.1 µM; Appendix Fig. S4C,D). This was reflected in a decrease of glutamate EC_{50} under UV light compared to under green light (Appendix Fig. S4E, see also Fig. EV2E). As expected, the photomodulation ratio measured at the UV steady-state current ($I_{UV, SS}$ / $I_{490 nm}$) displayed a clear glutamate-dependence (Appendix Fig. S4D). On the contrary, the photomodulation ratio measured at the UV peak current ($I_{UV, peak}$ / $I_{490 nm}$) was nearly independent of glutamate

concentration, with less than 10% difference of photomodulation ratios between the four glutamate concentrations tested (Appendix Fig. S4D).

We next investigated the glycine-dependence of photomodulation in presence of a saturating concentration of glutamate. Similarly to glutamate, UV induced a sustained potentiation for saturating (100 µM) and near-saturating (> 1 µM) concentrations of glycine but became transient at a glycine concentration lower than the EC_{50} (0.1 µM; Appendix Fig. S4F,G). Current relaxation was however much less marked than for glutamate, with a steady-state current under UV light representing 72% of the peak current for 0.1 µM glycine (Appendix Fig. S4H) compared to 50% of the peak current for 0.1 µM glutamate (Appendix Fig. S4E). This decrease in UV steady-state at low glycine concentrations was not translated into a significant change of glycine EC_{50} under UV light compared to 490 nm light (Appendix Fig. S4H). The photomodulation ratio measured at the UV peak slightly increased (by 20%) at low concentrations of glycine (Appendix Fig. S4G). In our *ex vivo* experiments, slices were perfused with 20 µM glycine so we can assume to be in a range of glycine concentrations for which the photomodulation ratio at the UV peak is constant.

Appendix Figures

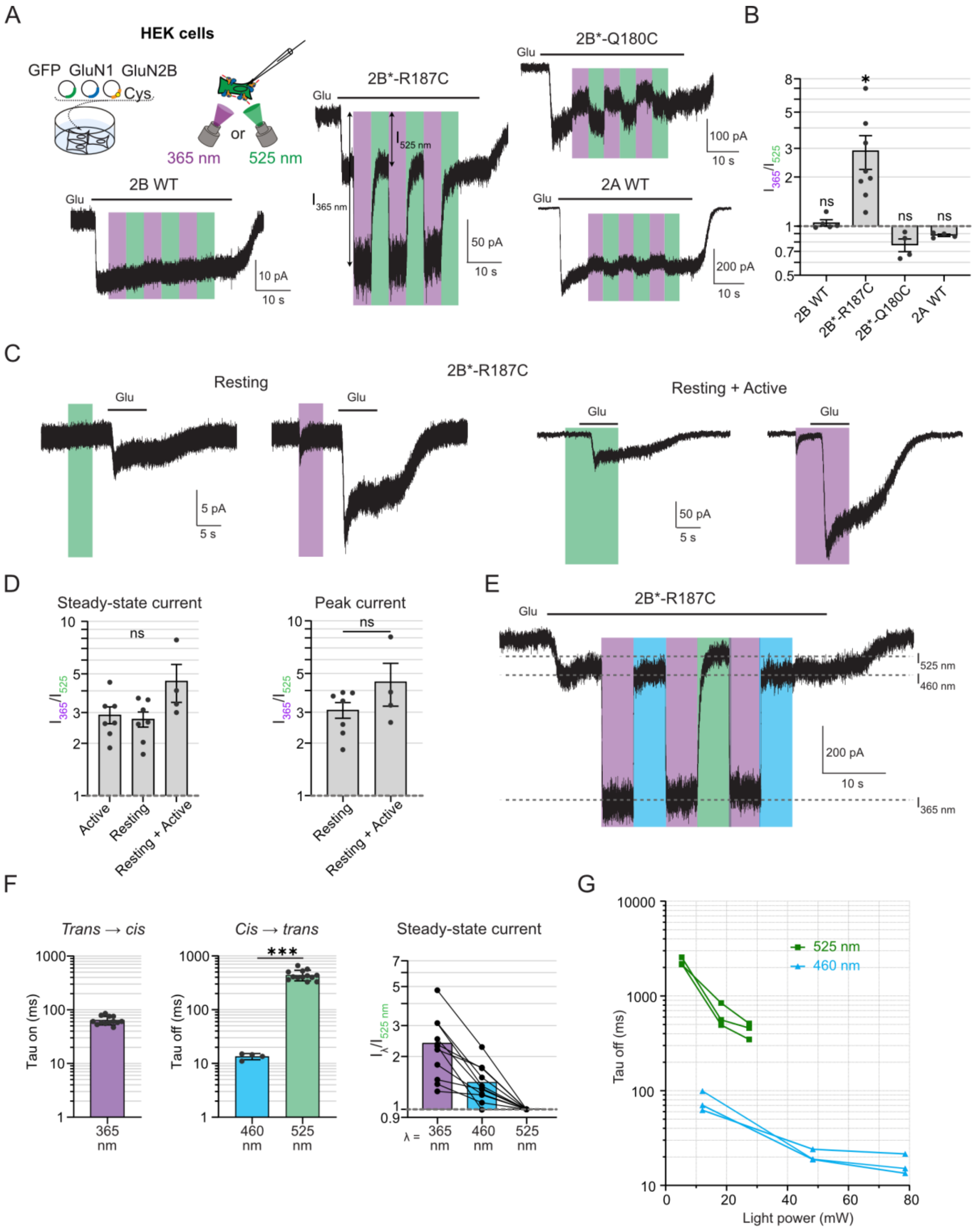

Appendix Fig. S1 (related to Fig. 2): Up to 3-fold potentiation of GluN2B diheteromer currents in HEK cells.

(A) Current traces from MASp-labeled HEK cells expressing GluN1/GluN2B WT, GluN1/GluN2B*-Q180C, GluN1/GluN2B*-R187C, and GluN1/GluN2A WT receptors, following application of glutamate (100 µM) under 365 nm (violet bars) or 525 nm (green bars) illumination. Cells were constantly perfused with glycine (100 µM). **(B)** Summary of the photomodulation ratios ($I_{365\ nm}$ / $I_{525\ nm}$) of currents from the different receptors in (A)

expressed in HEK cells. n.s., $p > 0.05$; **, $p < 0.01$; multiple one sample Wilcoxon tests against the value 1, p-values were adjusted for multiple comparisons using Bonferroni correction. Photomodulation values (mean ± s.e.m) and number of cells are indicated in Appendix Table S1. Average basal current values ($I_{525\ nm}$) are indicated in Appendix Table S4.

(C,D) No state-dependence of MASp-induced photomodulation. **(C)** GluN1/GluN2B*-R187C current traces in HEK cells under different illumination protocols. Left, light was applied before agonist application, allowing receptor modulation only from its resting state (Resting). Right, light was applied before and during agonist application, allowing receptor modulation from both its resting and activated states (Resting + Active). The small current peak observed at the onset of UV light in absence of agonist application corresponds to a transient potentiation of NMDARs tonically activated by small amounts of contaminating glutamate (see Appendix Fig. S4). **(D)** Photomodulation ratios of steady-state (left) and peak (right) currents following light application during the active state only (Active, panel a), the resting state only (Resting, panel c, left) and both the active and resting states (resting + Active, panel c, right). Photomodulation ratios under these three states were measured from HEK cells from the same coverslip (hence labeled at the same time) to minimize any variability linked to the labeling procedure. Steady-state current: Active condition, n = 7; Resting condition, n = 7; Resting + Active condition, n = 4. Peak current: Resting condition, n = 7; Resting + Active condition, n = 4. n.s., $p > 0.05$, Kruskall Wallis (left) and Mann Whitney (right) tests.

(E-G) Wavelength-dependence of MASp-induced photomodulation. **(E)** GluN1/GluN2B*-R187C current trace in a MASp-labeled HEK cell following illumination with UV (365 nm, violet bar), blue (460 nm, blue bar) or green (525 nm, green bar) light. Cells were pre-illuminated with blue light before current recording. **(F)** Time constants of current potentiation by UV light (left) and current recovery from potentiation by blue or green light (middle), and steady-state currents under the different light conditions relative to the current under green light, which represents the receptor basal activated state (right). Kinetics of photomodulation were measured at maximal light power through a 10x objective (~10 mW for 365 nm, 79 mW for 460 nm and 27 mW for 525 nm). Tau on (365 nm) = 64 ± 13 ms (n = 13), Tau off (460 nm) = 13.5 ± 0.9 ms (n = 4), Tau off (525 nm) = 442 ± 27 ms (n = 13). ***, $p < 0.001$, Mann Whitney test. Relative steady-state (SS) values: SS(365 nm) = 2.38 ± 0.30; SS(460 nm) = 1.43 ± 0.11; n = 11 for each condition. **(G)** Kinetics of current recovery from potentiation by blue or green light as a function of light power.

Recordings were all performed at pH 7.3, -60 mV. Data displayed as mean ± s.e.m. Exact p-values are summarized in Dataset EV1.

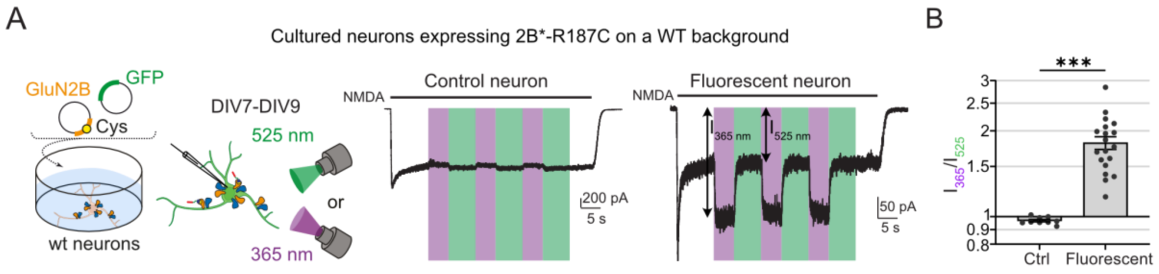

Appendix Fig. S2 (related to Fig. 2): Strong photomodulation of GluN2B*-R187C diheteromers in cultured cortical neurons.

(A) Left, expression of the GluN2B*-R187C subunit in cultured cortical neurons from wild-type animals through *ex utero* electroporation (see Methods). Right, current traces from DIV7-9 neurons labeled with MASp, following application of the selective NMDAR agonist NMDA (300 µM), under 365 nm (violet bars) or 525 nm (green bars) illumination. Cells were constantly perfused with D-serine (50 µM). Fluorescent neurons express the 2B*-R187C subunit, while non-fluorescent (control) neurons only express the endogenous NMDAR population. (**B**) Summary of photomodulation ratios of MASp-labeled, fluorescent and control (non-fluorescent) neurons. ***, $p < 0.001$, Mann Whitney test.

Recordings in cultured cortical neurons were performed at pH 7.3, -60 mV. Photomodulation values (mean ± s.e.m) and number of cells are summarized in Appendix Table S1. Average basal current values ($I_{525\ nm}$) are indicated in Appendix Table S5. Exact p-values are summarized in Dataset EV1.

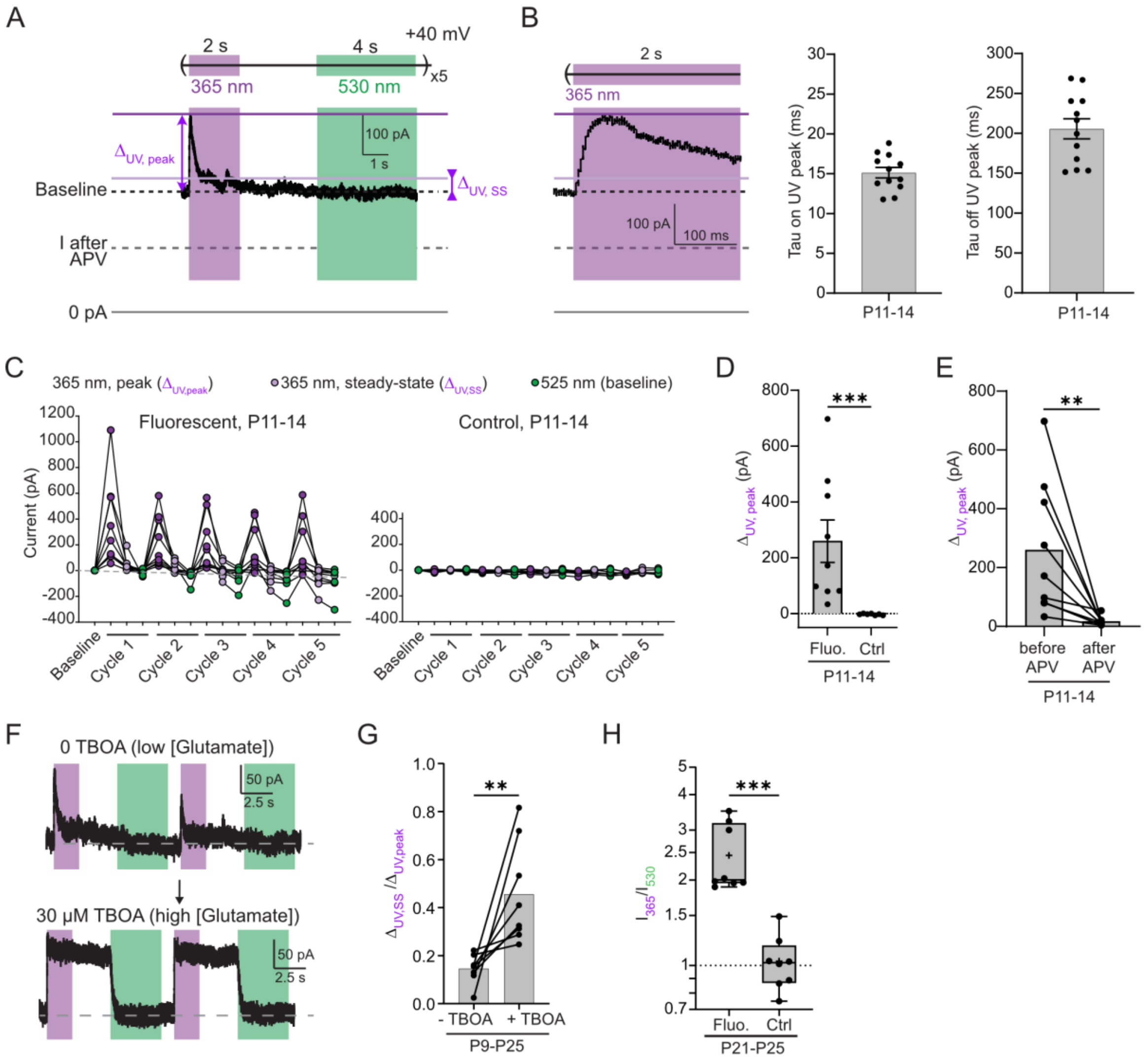

Appendix Fig. S3 (related to Fig. 3):

Unless otherwise noted, experiments were performed on slices from P11-14 electroporated animals. **(A)** NMDAR tonic current trace of the MASp-labeled, P13 fluorescent neuron shown in Fig. 3D displaying the different values used in the following panels. $\Delta_{UV,\ peak}$ represents the difference between the peak current amplitude at the onset of UV irradiation and the current amplitude under green light (baseline current). $\Delta_{UV,\ SS}$ represents the difference between the steady-state current amplitude at the end of UV irradiation and the current amplitude under green light (baseline current).

(B) Left, zoom on the UV-induced peak of the NMDA tonic current trace from (A). Middle and right, time constants of UV-induced potentiation (Tau on UV peak, left) and relaxation (Tau off UV peak, right). Tau on UV peak = 15.1 ± 0.7 ms, n = 12 and Tau off UV peak = 206 ± 13 ms, n = 12.

(C) Tonic current amplitudes of MASp-labeled, fluorescent (left) and control (right) neurons during the 5-cycle illumination protocol. See Panel (A) for annotations. n = 9 fluorescent neurons and 7 non-fluorescent (control) neurons.

(D) UV-induced increase of tonic current amplitude at peak ($\Delta_{UV, peak}$) for each MASp-labeled, fluorescent and control (non-fluorescent, denoted ctrl) neuron displayed in Panel C, and averaged over the 5 cycles of illumination. ***, $p < 0.001$, Mann Whitney test.

(E) APV suppresses UV-induced increase of tonic current at peak of MASp-labeled, fluorescent neurons. n = 9 cells. **, $p < 0.01$, Mann-Whitney test.

(F) Photomodulation of the tonic current of a MASp-labeled, P9 fluorescent neuron in basal conditions (0 TBOA, top) and after 10 min treatment of the slice with the glutamate transporter inhibitor TBOA (30 µM TBOA, bottom). As tonic glutamate concentration increases, UV-induced potentiation goes from transient to sustained.

(G) Relative amplitude of steady-state over peak UV-induced increase in tonic current ($\Delta_{UV, SS} / \Delta_{UV, peak}$) before (-TBOA) and after (+TBOA) TBOA treatment. Animals with ages ranging from P9 to P25 were used for this experiment. n = 8 cells. **, $p < 0.01$; Wilcoxon matched-pairs signed rank test.

(H) Photomodulation ratios of NMDA tonic currents of MASp-labeled, fluorescent and control (non-fluorescent) neurons from P21-P25 animals. n = 8 for each condition. Photomodulation values (mean ± s.e.m) and cell numbers are summarized in Appendix Table S6. ***, $p < 0.001$, Mann Whitney test. Box plots: centerlines show the median; crosses show the mean; box limits indicate the 25th and 75th percentiles; whiskers extend to the minimum and maximum.

All recordings in brain slices were performed at physiological pH. Data displayed as mean ± s.e.m. Exact p-values are summarized in Dataset EV1.

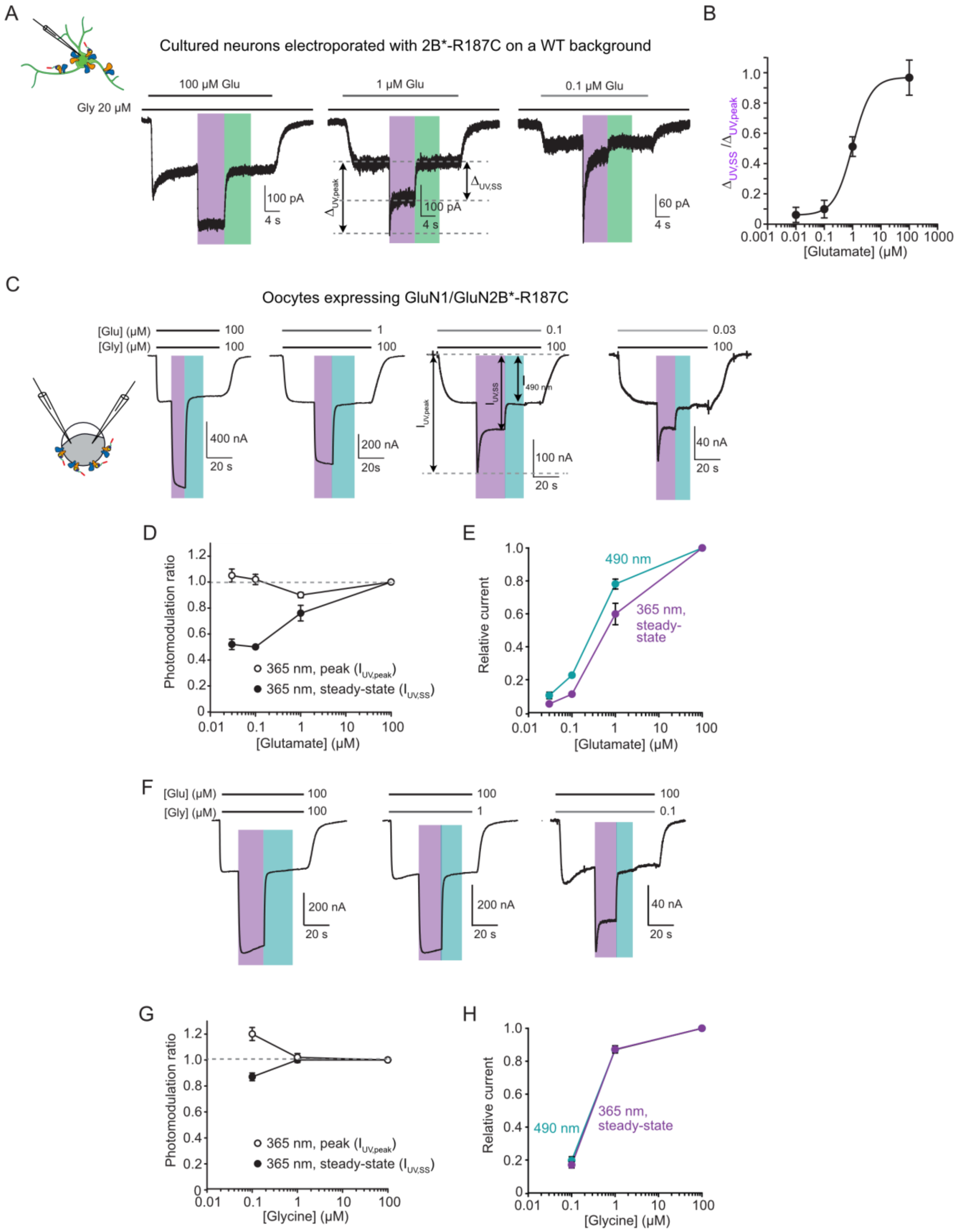

Appendix Fig. S4 (related to Fig. 3): Stability of Opto2B photoenhancement depends on glutamate concentration.

(A) Current traces from a cultured cortical neuron electroporated with the GluN2B*-R187C subunit and labeled with MASp showing transient UV (violet bar)-induced potentiation of NMDAR currents under non-saturating glutamate (Glu) concentrations (0.1 and 1 μM), while UV potentiation was stable at saturating glutamate concentration (100 μM). NMDAR currents were isolated using 10 μM NBQX, 100 μM picrotoxin and 10 μM strychnine, and 20 μM

glycine was applied throughout the experiment (same conditions as in *ex vivo* experiments in Fig. 3). The experimental strategy to express the mutated GluN2B subunit on a WT background was the same as in Appendix Fig. S2A.

(B) The ratio of steady-state over peak current ($\Delta_{UV, SS}$ / $\Delta_{UV, peak}$; see Panel (A) for measurement of $\Delta_{UV, SS}$ and $\Delta_{UV, peak}$) increases as glutamate concentration increases. n = 6-9 cells. Recordings were performed at pH 7.3, -60 mV.

(C-H) Experiments were performed in Xenopus oocytes expressing GluN1/GluN2B*-R187C at pH 6.5, -60 mV. **(C-E)** Glutamate dependence of GluN2B diheteromer photomodulation. **(C)** Current traces from an oocyte expressing GluN1/GluN2B*-R187C and labeled with MASp following application of varying concentrations of glutamate together with a saturating concentration (100 µM) of glycine and under 365 (violet bar) or 490 nm (blue-green bar) illumination. **(D)** While the photomodulation ratios at steady-state ($I_{UV, SS}$ / $I_{490\ nm}$, black dots) are highly dependent on glutamate concentration, the photomodulation ratios calculated from the UV peak currents ($I_{UV, peak}$ / $I_{490\ nm}$, white dots) were almost independent of glutamate concentration (they varied by less than 10% across all glutamate concentrations). For each cell, $I_{UV, peak}$ was measured at the time of peak measured for 0.1 µM glutamate, i.e. ~0.9 s after the onset of UV illumination. $I_{UV, SS}$ was measured at the end of the UV illumination step. $I_{490\ nm}$ was measured at the end of the 490 nm illumination step. n = 4-6 cells. **(E)** Relative currents at different glutamate concentrations normalized to the current at 100 µM glutamate (saturating concentration). n = 4-6 cells.

(F-H) Glycine dependence of GluN1/GluN2B photomodulation. **(F)** Current traces from an oocyte expressing GluN1/GluN2B*-R187C and labeled with MASp following application of varying concentrations of glycine together with a saturating concentration (100 µM) of glutamate and under 365 (violet bar) or 490 nm (blue-green bar) illumination. **(G)** UV potentiation was stable for 100 and 1 µM glycine but became transient for 0.1 µM glycine. Maximum UV potentiation ($I_{UV, peak}$ / $I_{490\ nm}$, white dots) increased by 20% at low glycine concentrations compared to higher 1 and 100 µM concentrations. n = 7 cells. **(H)** Relative currents at different glycine concentrations normalized to the current at 100 µM glycine (saturating concentration) (n = 7 cells).

All data are displayed as mean ± s.e.m.

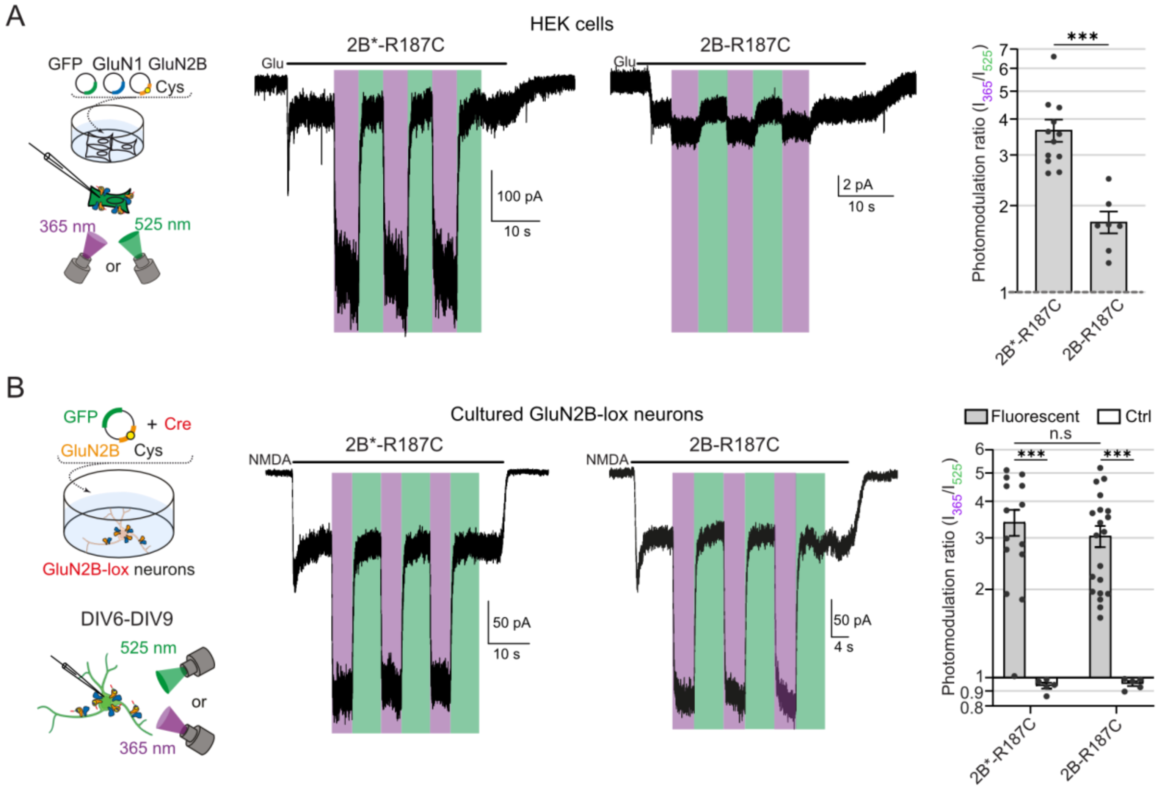

Appendix Fig. S5 (related to Fig. 4): Design strategy of the Opto2B mouse line: neutralizing the endogenous cysteine C395 is not necessary to obtain strong photomodulation in native preparations.

(A) Left, current traces from HEK cells expressing GluN1/GluN2B-R187C-C395S (GluN1/GluN2B*-R187C) and GluN1/GluN2B-R187C receptors (with endogenous cysteine 395 intact), and labeled with MASp, following application of glutamate (100 µM) under 365 nm (violet bars) or 525 nm (green bars) illumination. Cells were constantly perfused with glycine (100 µM). Right, summary of the photomodulation ratios ($I_{365\ nm}$ / $I_{525\ nm}$) for the different GluN2B mutants expressed in HEK cells. Note that the photomodulation ratio is much lower for GluN1/GluN2B-R187C (1.75 ± 0.15, n = 7) compared to GluN1/GluN2B*-R187C (3.66 ± 0.32, n = 12). Photomodulation values (mean ± s.e.m) and number of cells are summarized in Appendix Table S1. Average basal current values ($I_{525\ nm}$) are indicated in Appendix Table S4. ***, p < 0.001; Mann Whitney test.

(B) Left, current traces from DIV6-9 cortical neurons expressing GluN1/GluN2B-R187C-C395S (GluN1/GluN2B*-R187C) and GluN1/GluN2B-R187C receptors, and labeled with MASp, following application of NMDA (300 µM) under 365 nm (violet bars) or 525 nm (green bars) illumination. Cells were constantly perfused with D-serine (50 µM). In this particular experiment, we adopted a strategy of molecular replacement of the endogenous, WT GluN2B subunits by the mutated GluN2B subunits using the Cre-lox approach. Dissociated cortical neurons from floxed-Grin2B mice (von Engelhardt *et al*, 2008) were electroporated with pIRES plasmids coding for GFP and a mutated GluN2B subunit (Sanz-Clemente *et al*, 2013),

and plasmids coding for the Cre recombinase (to remove endogenous GluN2B expression) and for the fluorescent marker TdTomato (see Methods). Right, summary of the photomodulation ratios ($I_{365\ nm}$ / $I_{525\ nm}$) of currents from the different GluN2B mutants expressed in cultured neurons. Note that, contrary to what was observed on HEK cells, the photomodulation ratio is similar between GluN1/GluN2B-R187C (3.04 ± 0.25, n = 20) and GluN1/GluN2B*-R187C (3.25 ± 0.35, n = 15). n.s, p > 0.05; ***, p < 0.001; Multiple Mann-Whitney tests, p-values were adjusted for multiple comparisons using Bonferroni correction. Only the pre-selected indicated comparisons were performed. Photomodulation values (mean ± s.e.m) and number of cells are summarized in Appendix Table S1. Average basal current values ($I_{525\ nm}$) are indicated in Appendix Table S5.

All recordings were performed at pH 7.3, -60 mV. Data displayed as mean ± s.e.m. Exact p-values are summarized in Dataset EV1.

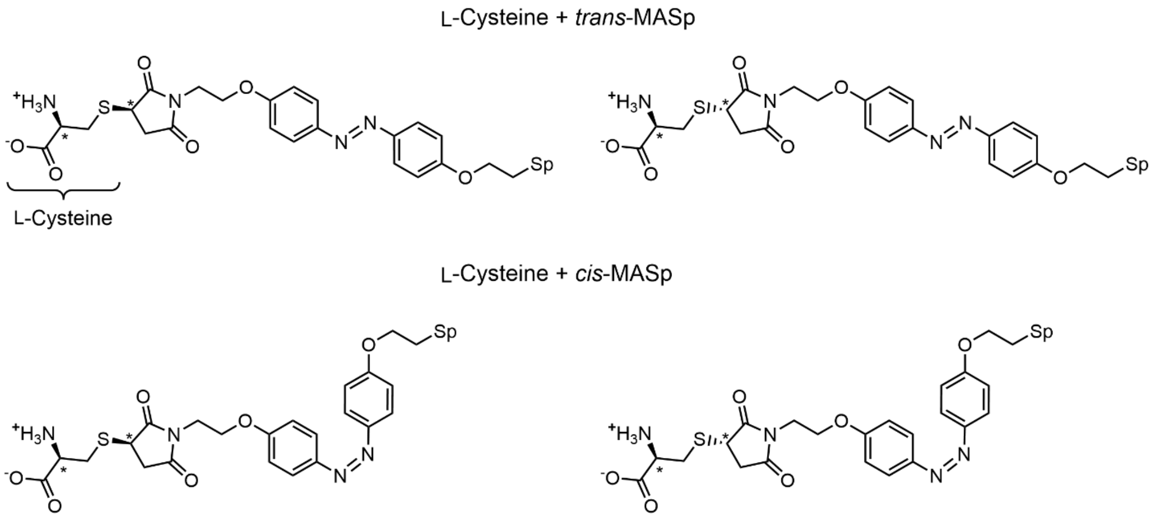

Appendix Fig. S6: Chemical structures of the four diastereoisomeric products of the reaction between MASp and L-Cysteine. Stars indicate asymmetric carbons.

Appendix Tables

Appendix Table S1: Summary of *in vitro* photomodulation data. Photomodulation steady-state values of different NMDAR constructs labeled with MASp in Xenopus oocytes, HEK cells and cultured cortical neurons. Values as mean ± s.e.m. Cell numbers in parentheses.

**Construct**	I_{UV} / I_{green}[a]			
	**Oocytes (pH 6.5)**	**Oocytes (pH 7.3)**	**HEK cells (pH 7.3)**	**Neurons (pH 7.3)**
**2B WT**	1.00 ± 0.02 (n = 20)	0.99 ± 0.01 (n = 4)	1.048 ± 0.005 (n = 5)	- 0.968 ± 0.009 (n = 9) (non-fluorescent neurons on WT background; Appendix Fig. S2)<br><br>- 0.95 ± 0.02 (n = 5) (2B*-R187C condition) 0.94 ± 0.02 (n = 5) (2B-R187C condition) (non-fluorescent neurons ; molecular replacement strategy ; Appendix Fig. S5B)
**2B*-Q180C**	0.39 ± 0.02 (n = 25)	0.84 ± 0.08 (n = 13)	0.77 ± 0.07 (n = 4)	
**2B*-R187C**	3.53 ± 0.21 (n = 32)	1.97 ± 0.17 (n = 21)	2.9 ± 0.7 (n = 8) (Appendix Fig. S1)<br><br>3.7 ± 0.3 (n = 12) (Appendix Fig. S5A)	- 1.81 ± 0.09 (n = 19) (fluorescent neurons on WT background; Appendix Fig. S2)<br><br>- 3.40 ± 0.35 (n = 14) (fluorescent neurons ; molecular replacement strategy ; Appendix Fig. S5B)
**2B-R187C**			1.75 ± 0.40 (n = 7)	- 3.04 ± 0.25 (n = 20) (fluorescent neurons ; Cre-lox strategy)
**2A WT**	0.92 ± 0.02 (n = 22)		0.88 ± 0.02 (n = 4)	
**2C WT**	1.02 ± 0.01 (n = 5)			
**2D WT**	1.00 ± 0.01 (n = 5)			
**2B*-R187C-r1 / 2B*-R187C-r2**	3.07 ± 0.22 (n = 9)			
**2B-r1 / 2B*-R187C-r2**	1.21 ± 0.02 (n = 14)			
**2A-r1 / 2B*-R187C-r2**	1.01 ± 0.01 (n = 17)			

[a]Green light is 490 nm for oocytes and 525 nm for HEK cells and neurons.

Appendix Table S2: **Summary of several pharmacological parameters of 2B*-Q180C and 2B*-R187C mutants unlabeled (- MASp) and labeled (+ MASp) with MASp under different illumination conditions.** Values as mean ± s.e.m. Cell numbers in parentheses.

		**Glu EC_{50}**	**Gly EC_{50}**	**Relative Po[a]**	**$I_{pH\ 7.3}$ / $I_{pH\ 6.5}$**	**$I_{spermine}$ / I_0[b]**
**2B wt**	**- MASp**	1.44 ± 0.15 (n = 9)	0.48 ± 0.09 (n = 4)	1.01 ± 0.04 (n = 15)	10.4 ± 0.3 (n = 20)	8.0 ± 0.4 (n = 21)
**2B*-Q180C**	**- MASp**	1.43 ± 0.10 (n = 3)	0.29 ± 0.03 (n = 5)	0.96 ± 0.1 (n = 8)	9.5 ± 0.6 (n = 11)	7.5 ± 0.5 (n = 11)
	**+ MASp, 365 nm**	1.83 ± 0.12 (n = 7)	0.29 ± 0.06 (n = 3)	0.76 ± 0.14 (n = 7)	9.5 ± 0.8 (n = 6)	4.3 ± 0.4 (n = 9)
	**+ MASp, 490 nm**	2.49 ± 0.11 (n = 6)	0.57 ± 0.08 (n = 6)	2.15 ± 0.04 (n = 9)	5.2 ± 0.3 (n = 5)	2.7 ± 0.2 (n = 8)
**2B*-R187C**	**- MASp**	0.94 ± 0.04 (n = 11)	0.49 ± 0.05 (n = 8)	0.64 ± 0.04 (n = 6)	13.4 ± 0.6 (n = 9)	11.5 ± 0.8 (n = 9)
	**+ MASp, 365 nm**	1.83 ± 0.08 (n = 7)	0.30 ± 0.03 (n = 4)	2.22 ± 0.19 (n = 5)	6.9 ± 0.5 (n = 7)	4.3 ± 0.4 (n = 7)
	**+ MASp, 490 nm**	0.75 ± 0.07 (n = 8)	0.43 ± 0.05 (n = 4)	0.56 ± 0.04 (n = 5)	9.5 ± 0.5 (n = 7)	7.5 ± 0.7 (n = 7)

[a]Relative Po is measured as the rate of MK-801 inhibition normalized to the average rate of inhibition of wt GluN2B-NMDARs measured the same experimental day.
[b]Spermine was applied at a concentration of 200 µM (around its EC_{50}, see (Mony *et al*, 2011). Glu, glutamate; Gly, glycine.

Appendix Table S3: Average current values for MASp-labeled NMDAR constructs expressed in Xenopus oocytes. Values as mean ± s.e.m; cell numbers in parentheses.

**Construct**	**Basal current[a] (µA)**
2B WT	0.88 ± 0.18 (n = 20)
2B*-Q180C	0.63 ± 0.13 (n = 25)
2B*-R187C	0.34 ± 0.13 (n = 32)
2A WT	1.30 ± 0.24 (n = 22)
2C WT	0.33 ± 0.12 (n = 5)
2D WT	0.14 ± 0.03 (n = 5)
2B*-R187C-r1 / 2B*-R187C-r2	0.043 ± 0.01 (n = 9)
2B-r1 / 2B*-R187C-r2	0.34 ± 0.12 (n = 14)
2A-r1 / 2B*-R187C-r2	0.25 ± 0.11 (n = 17)

[a]Basal current was measured under 490 nm illumination, except for 2B*-Q180C, which was measured under 365 nm illumination (since this construct is potentiated by 490 nm light and in its basal state under UV illumination; see Fig. 1)

Appendix Table S4: Average current values for MASp-labeled NMDAR constructs expressed in HEK cells. Values as mean ± s.e.m; cell numbers in parentheses.

**Construct**	**Basal current[a] (pA)**
2B WT	36 ± 23 (n = 5)
2B-R187C-C395S (App. Fig. S1)	73 ± 17 (n = 8)
2B-Q180C-C395S	50 ± 10 (n = 4)
2A WT	218 ± 81 (n = 4)
2B-R187C-C395S (App. Fig. S5A)	20 ± 5 (n = 12)
2B-R187C (App. Fig. S5A)	29 ± 13 (n = 7)

[a]Basal current was measured under 525 nm illumination.

Appendix Table S5: Average current values for MASp-labeled, electroporated and non-electroporated, cultured cortical neurons. Values as mean ± s.e.m; cell numbers in parentheses.

**Condition**	**Basal current[a] (pA)**
Non-electroporated neuron on WT background (App. Fig. S2)	402 ± 131 (n = 9)
2B-R187C-C395S on WT background (App. Fig. S2)	408 ± 93 (n = 19)
2B-R187C-C395S on floxed-GluN2B background (App. Fig. S5B)	160 ± 40 (n = 14)
2B-R187C on floxed-GluN2B background (App. Fig. S5B)	99 ± 13 (n = 20)

[a]Basal current was measured under 525 nm illumination

Appendix Table S6: Summary of *ex vivo* photomodulation data on cortical pyramidal neurons. Values as mean ± s.e.m; cell numbers in parentheses.

**Photomodulation ($I_{365\ nm}$ / $I_{530\ nm}$)**		**Opto2B neurons**	**Control neurons**
**P11-14**	**NMDA EPSCs**	1.45 ± 0.04 (n = 9)	0.99 ± 0.02 (n = 7)
	**NMDA tonic current**	3.83 ± 0.45 (n = 9)	0.95 ± 0.03 (n = 7)
**P21-25**	**NMDA EPSCs**	1.27 ± 0.03 (n = 9)	1.01 ± 0.02 (n = 7)
	**NMDA tonic current**	2.44 ± 0.24 (n = 8)	1.04 ± 0.08 (n = 8)

Appendix Table S7: Summary of *ex vivo* photomodulation data in hippocampal CA1 pyramidal neurons. Values as mean ± s.e.m; cell numbers in parentheses.

**Photomodulation ($I_{365\ nm}$ / $I_{530\ nm}$)**		**WT**	**Opto2B**	**Opto2B / GluN2A KO**
**P5**	**NMDA EPSCs**		1.56 ± 0.17 (n = 6)	
	**NMDA tonic current**		3.53 ± 0.55 (n = 8)	3.05 ± 0.47 (n = 6)
**P8-12**	**NMDA EPSCs**	1.02 ± 0.04 (n = 10)	1.41 ± 0.06 (n = 20)	2.07 ± 0.20 (n = 10)
	**NMDA tonic current**	0.98 ± 0.04 (n = 4)	3.30 ± 0.39 (n = 15)	2.53 ± 0.13 (n = 12)
**P20-23**	**NMDA EPSCs**		1.28 ± 0.04 (n = 14)	1.63 ± 0.06 (n = 22)
	**NMDA tonic current**		1.46 ± 0.08 (n = 10)	2.12 ± 0.20 (n = 15)
**P37-47**	**NMDA EPSCs**		1.14 ± 0.03 (n = 14)	1.51 ± 0.05 (n = 17)
	**NMDA tonic current**		1.32 ± 0.12 (n = 8)	2.67 ± 0.42 (n = 14)

Appendix Table S8: Average NMDA-EPSC values in hippocampal CA1 pyramidal neurons. Values as mean ± s.e.m; cell numbers in parentheses

**Basal NMDA EPSCs (pA)**	**WT**	**Opto2B**	**Opto2B / GluN2A KO**
**P5**		89 ± 17 (n = 6)	
**P8-12**	156 ± 9 (n = 10)	158 ± 14 (n = 20)	132 ± 14 (n = 10)
**P20-23**		140 ± 21 (n = 14)	106 ± 12 (n = 22)
**P37-47**		144 ± 21 (n = 14)	133 ± 12 (n = 17)

Appendix References

von Engelhardt J, Doganci B, Jensen V, Hvalby Ø, Göngrich C, Taylor A, Barkus C, Sanderson DJ, Rawlins JNP, Seeburg PH, *et al* (2008) Contribution of hippocampal and extra-hippocampal NR2B-containing NMDA receptors to performance on spatial learning tasks. *Neuron* 60: 846–860

Mony L, Zhu S, Carvalho S & Paoletti P (2011) Molecular basis of positive allosteric modulation of GluN2B NMDA receptors by polyamines. *EMBO J* 30: 3134–3146

Sanz-Clemente A, Nicoll RA & Roche KW (2013) Diversity in NMDA receptor composition: many regulators, many consequences. *Neuroscientist* 19: 62–75
