# Supplementary figures and images for: Optochemical profiling of NMDAR molecular diversity at synaptic and extrasynaptic sites
Source: EMBO J. 2025 Jul 8;44(16):4577–610. doi: 10.1038/s44318-025-00498-x (PMC12361563; doi:10.1038/s44318-025-00498-x)

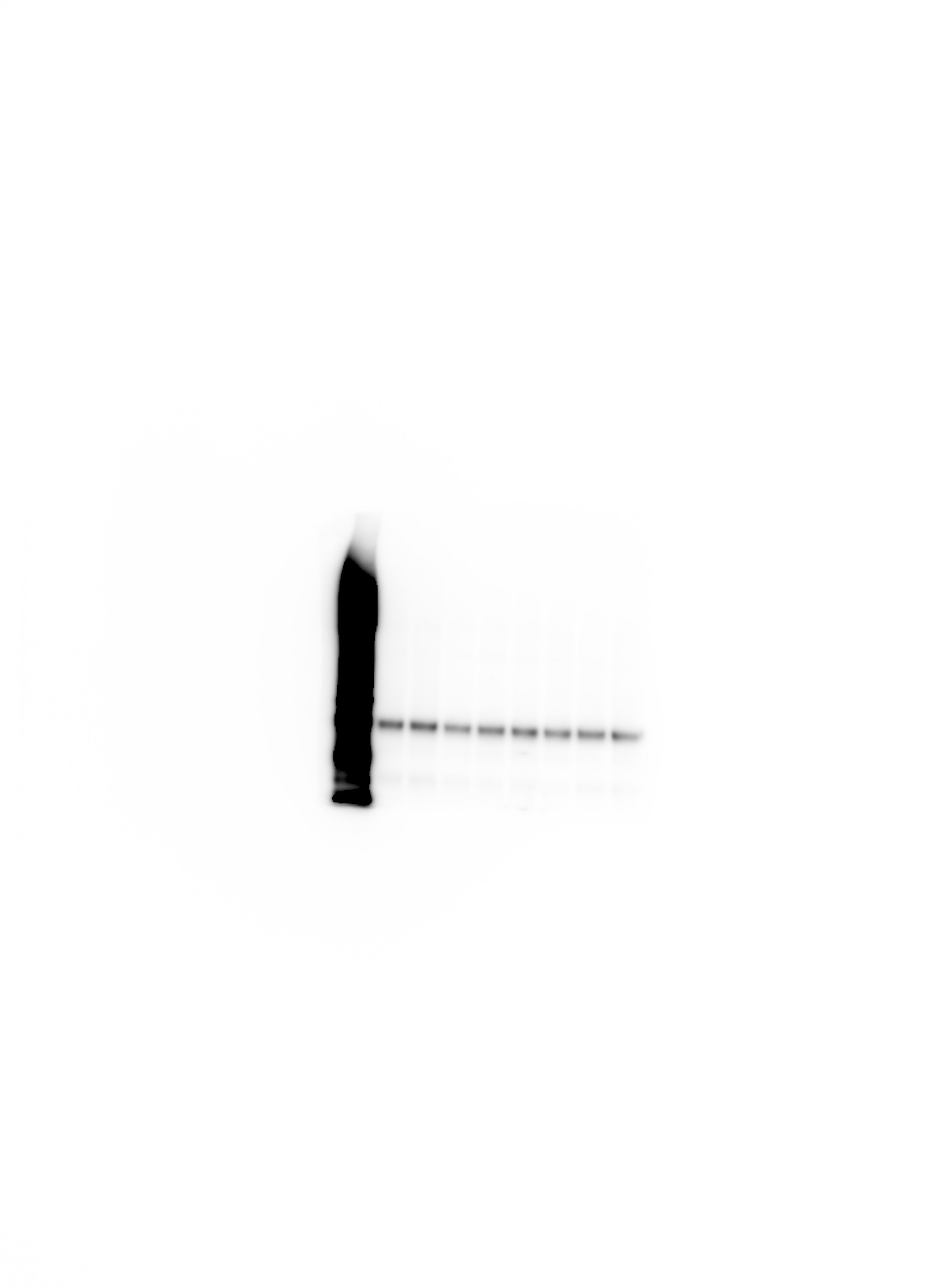

Supplement: Supplementary file 9 — Appendix + EV source data [file 44318_2025_498_MOESM9_ESM.zip › SD_EV_Appendix/EMBOJ-2024-119900_FigEV4_Westernblot_files/GluA1/Blot1_GluA1.tif]

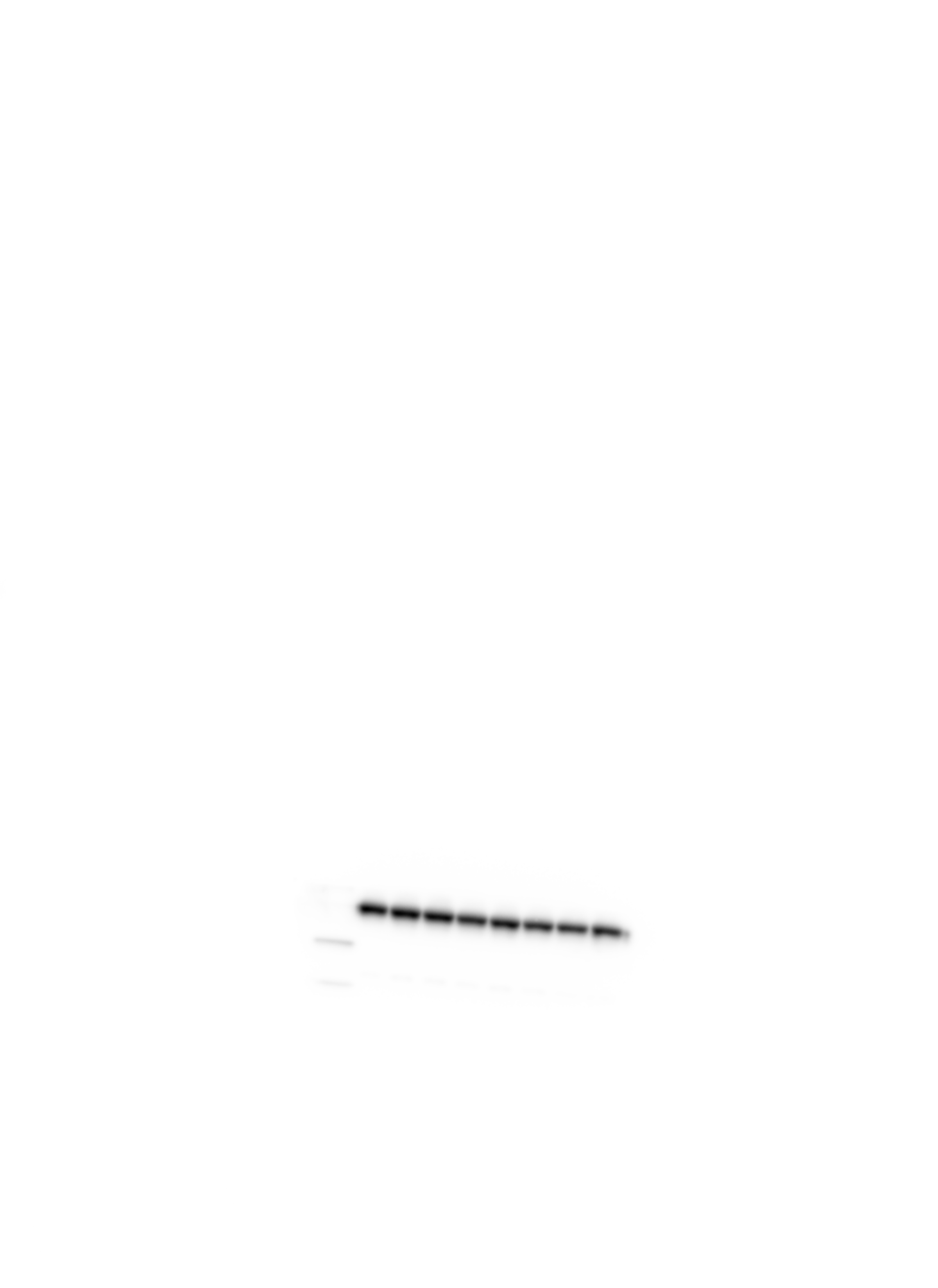

Supplement: Supplementary file 9 — Appendix + EV source data [file 44318_2025_498_MOESM9_ESM.zip › SD_EV_Appendix/EMBOJ-2024-119900_FigEV4_Westernblot_files/GluA1/Blot1_tubulinforGluA1.tif]

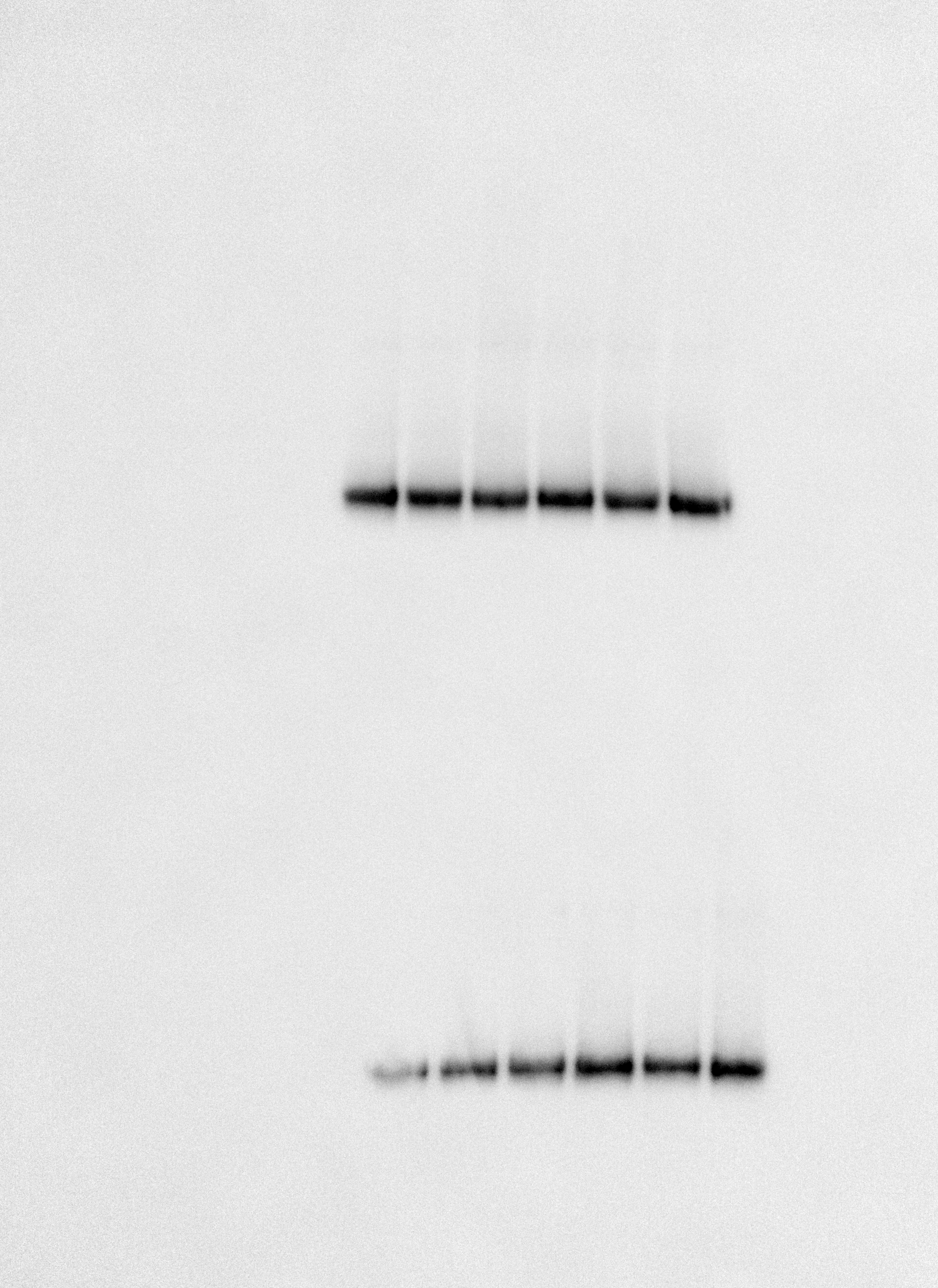

Supplement: Supplementary file 9 — Appendix + EV source data [file 44318_2025_498_MOESM9_ESM.zip › SD_EV_Appendix/EMBOJ-2024-119900_FigEV4_Westernblot_files/GluA1/Blot2_GluA1.tif]

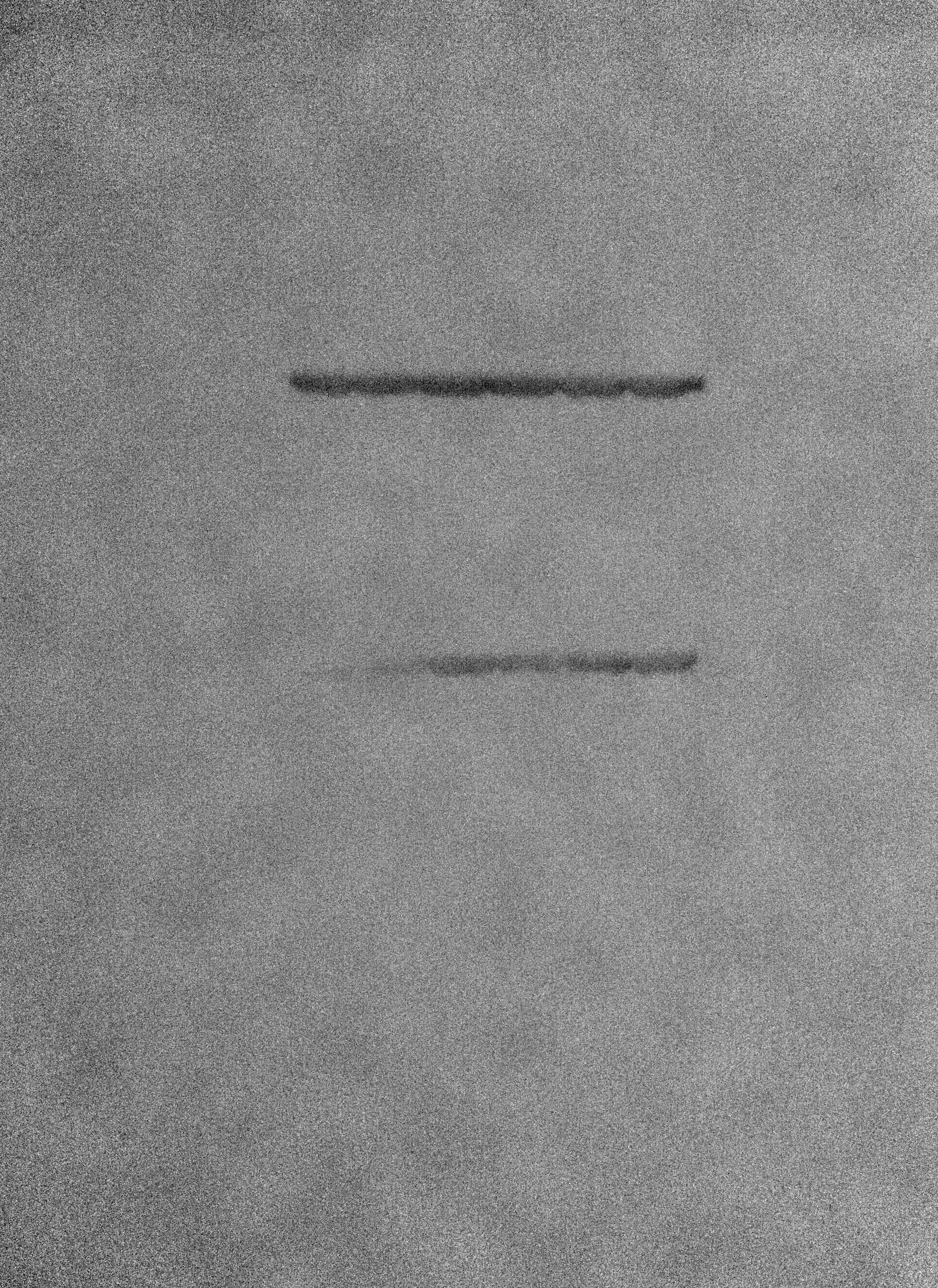

Supplement: Supplementary file 9 — Appendix + EV source data [file 44318_2025_498_MOESM9_ESM.zip › SD_EV_Appendix/EMBOJ-2024-119900_FigEV4_Westernblot_files/GluA1/Blot2_tubulinforGluA1.tif]

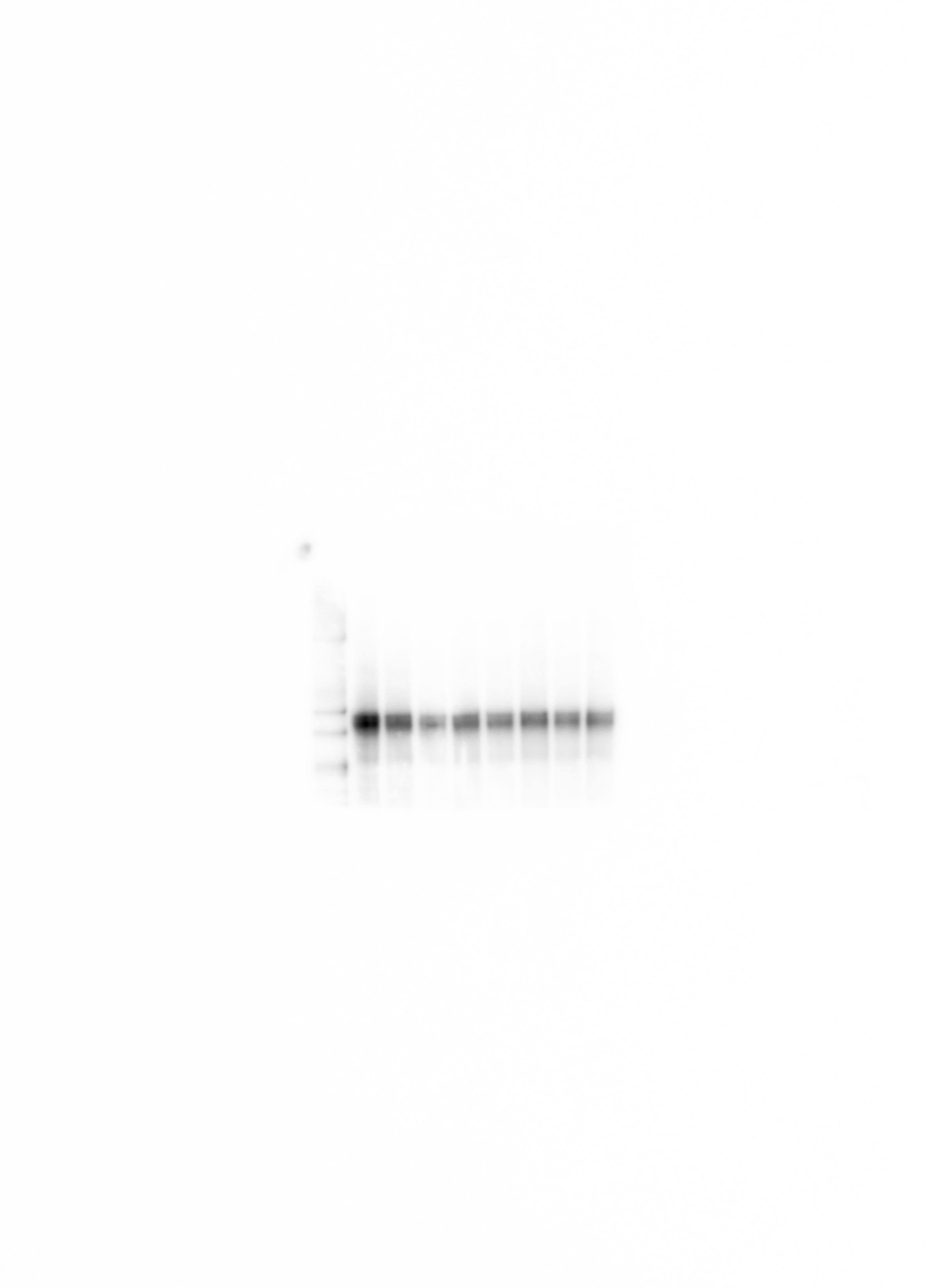

Supplement: Supplementary file 9 — Appendix + EV source data [file 44318_2025_498_MOESM9_ESM.zip › SD_EV_Appendix/EMBOJ-2024-119900_FigEV4_Westernblot_files/GluN1/Blot1_GluN1.tif]

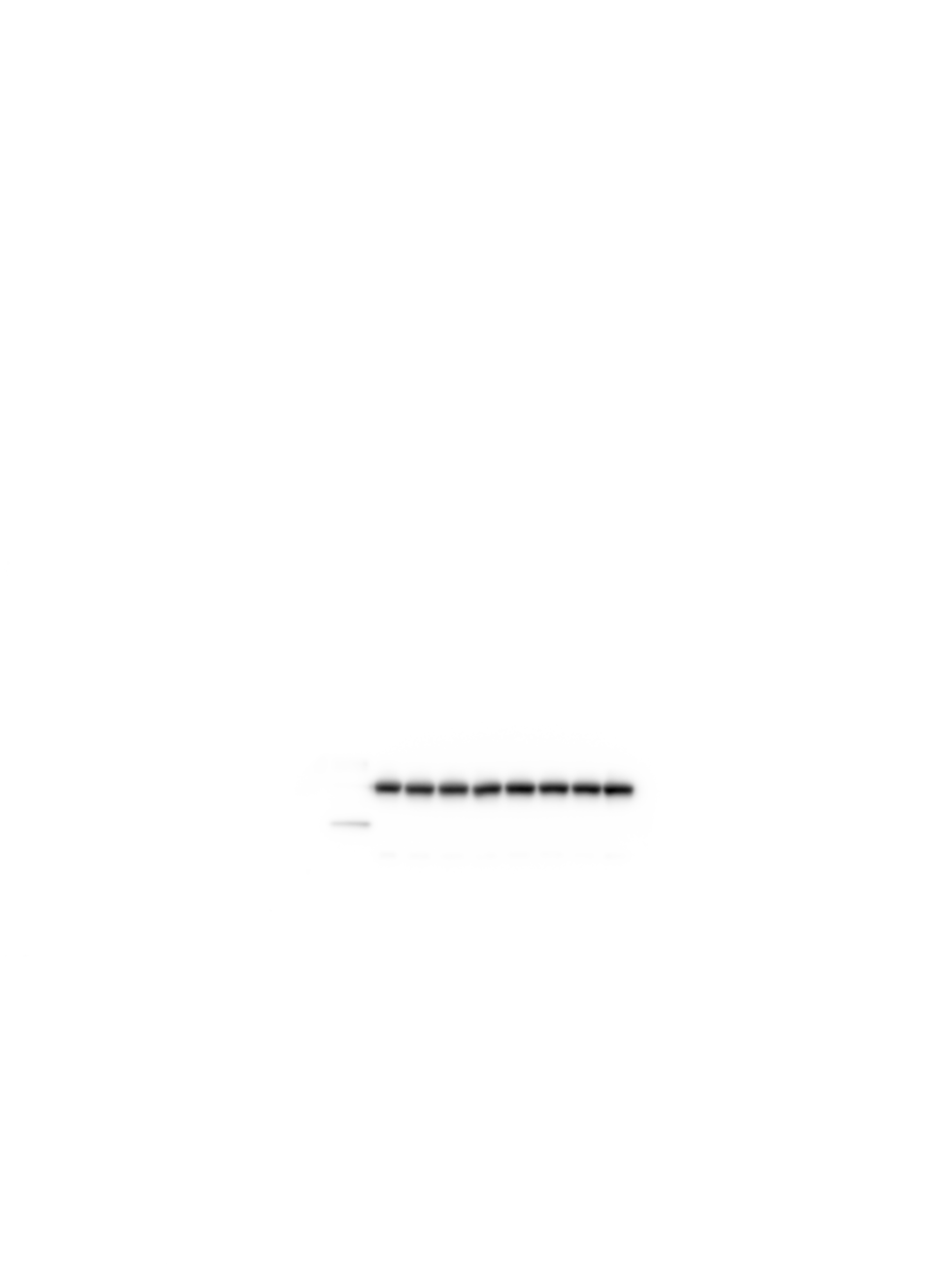

Supplement: Supplementary file 9 — Appendix + EV source data [file 44318_2025_498_MOESM9_ESM.zip › SD_EV_Appendix/EMBOJ-2024-119900_FigEV4_Westernblot_files/GluN1/Blot1_tubulinforGluN1.tif]

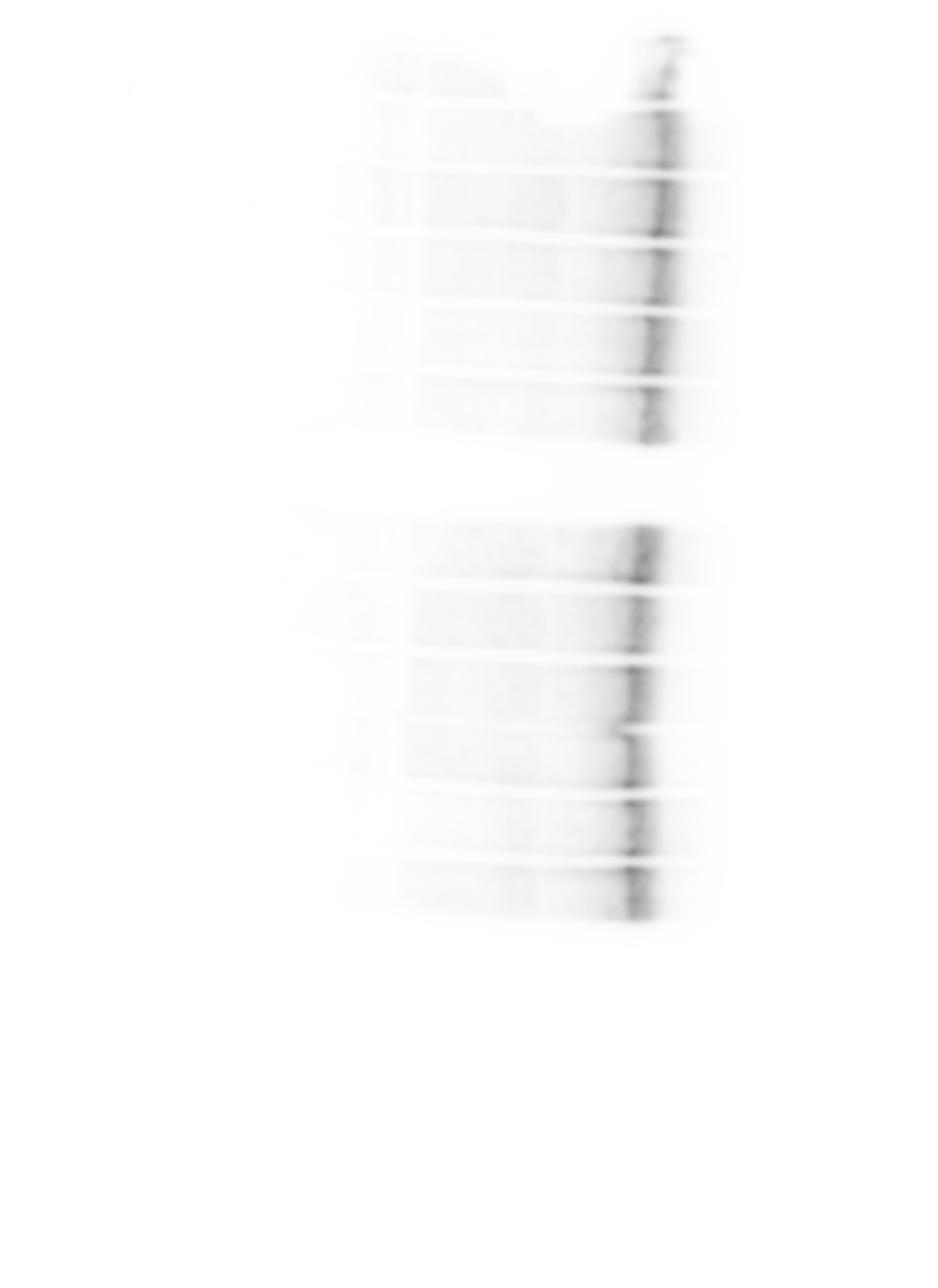

Supplement: Supplementary file 9 — Appendix + EV source data [file 44318_2025_498_MOESM9_ESM.zip › SD_EV_Appendix/EMBOJ-2024-119900_FigEV4_Westernblot_files/GluN1/Blot2_GluN1.tif]

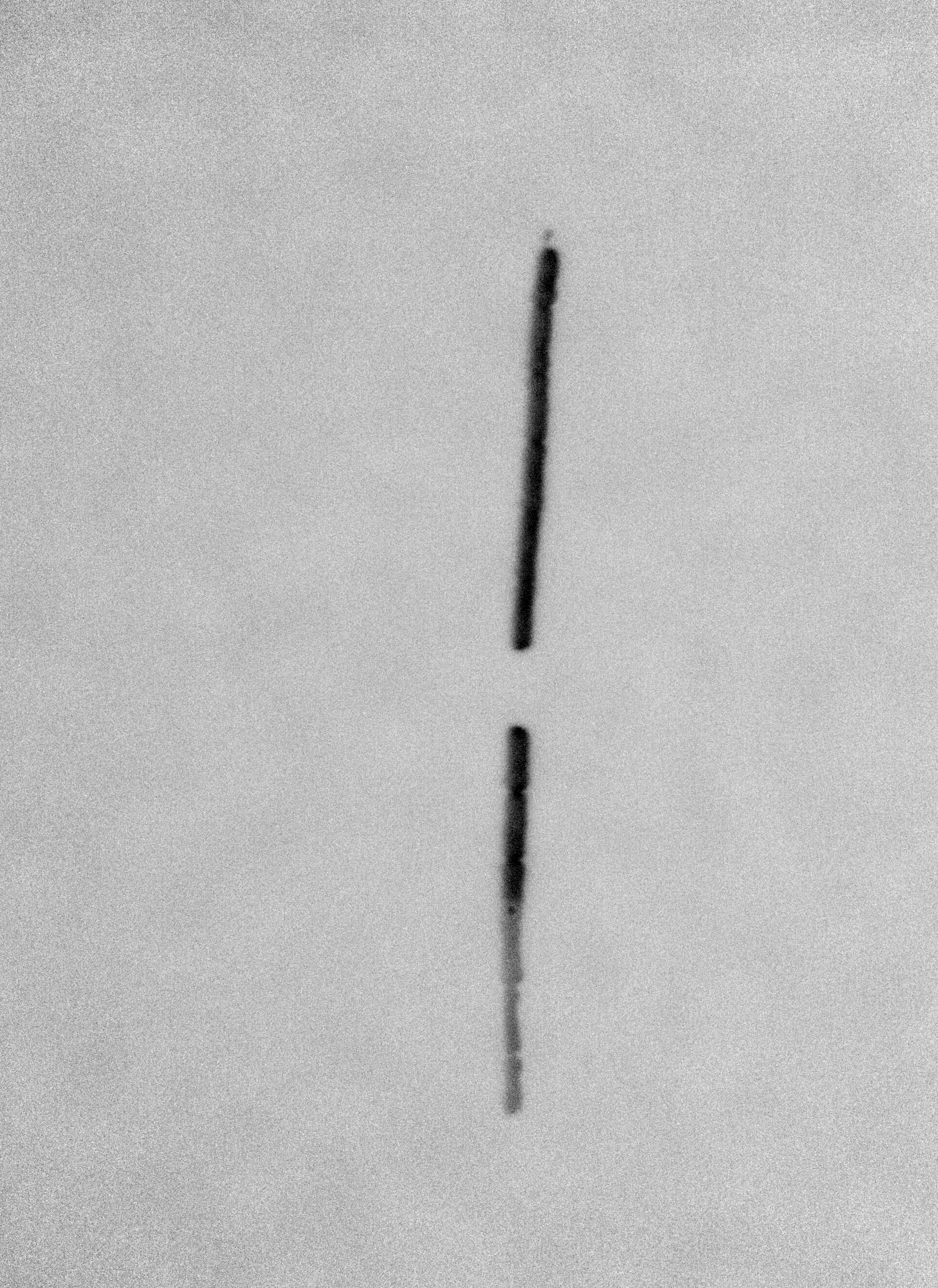

Supplement: Supplementary file 9 — Appendix + EV source data [file 44318_2025_498_MOESM9_ESM.zip › SD_EV_Appendix/EMBOJ-2024-119900_FigEV4_Westernblot_files/GluN1/Blot2_tubulinforGluN1.tif]

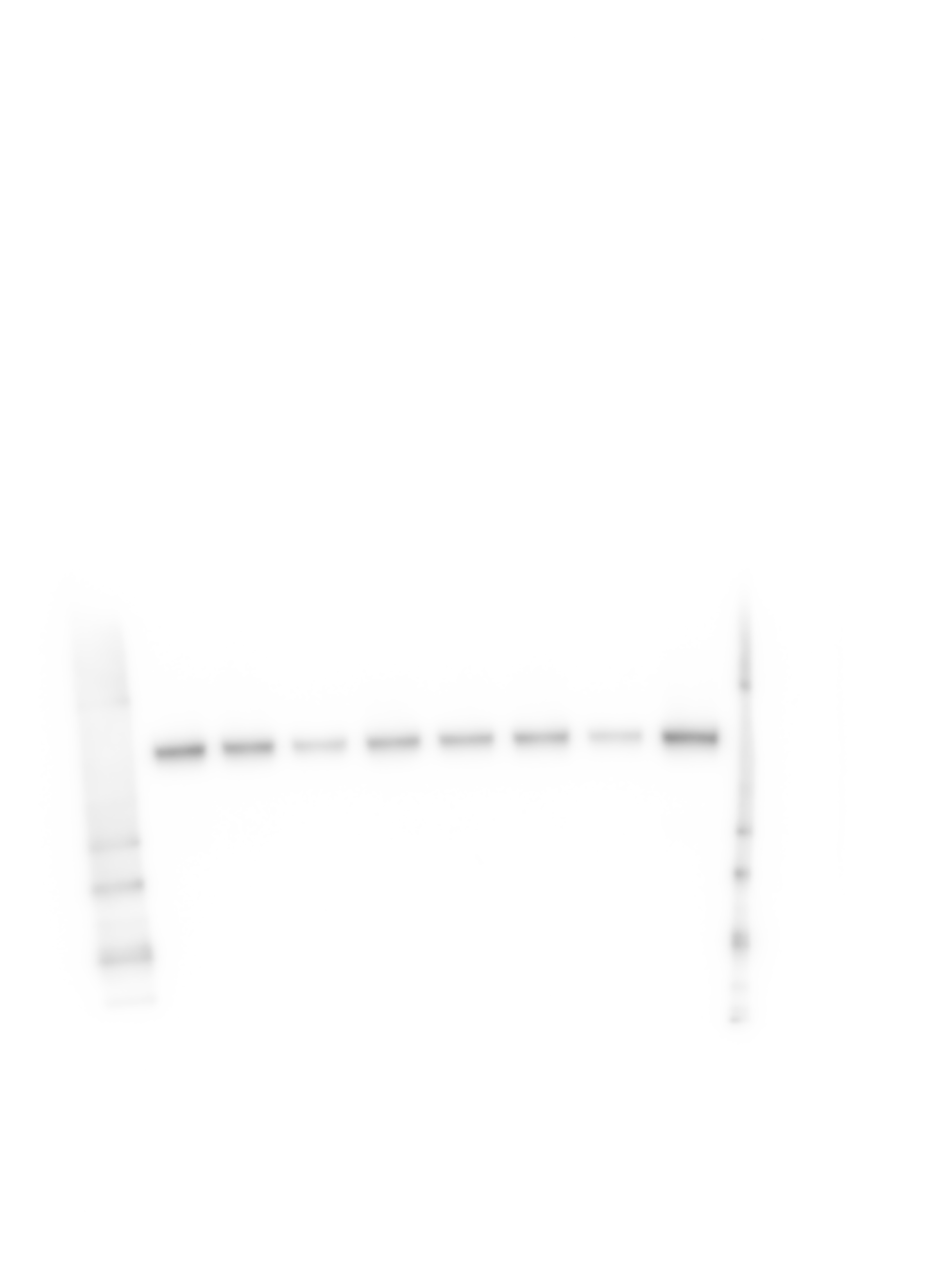

Supplement: Supplementary file 9 — Appendix + EV source data [file 44318_2025_498_MOESM9_ESM.zip › SD_EV_Appendix/EMBOJ-2024-119900_FigEV4_Westernblot_files/GluN2A/Blot1_GluN2A.tif]

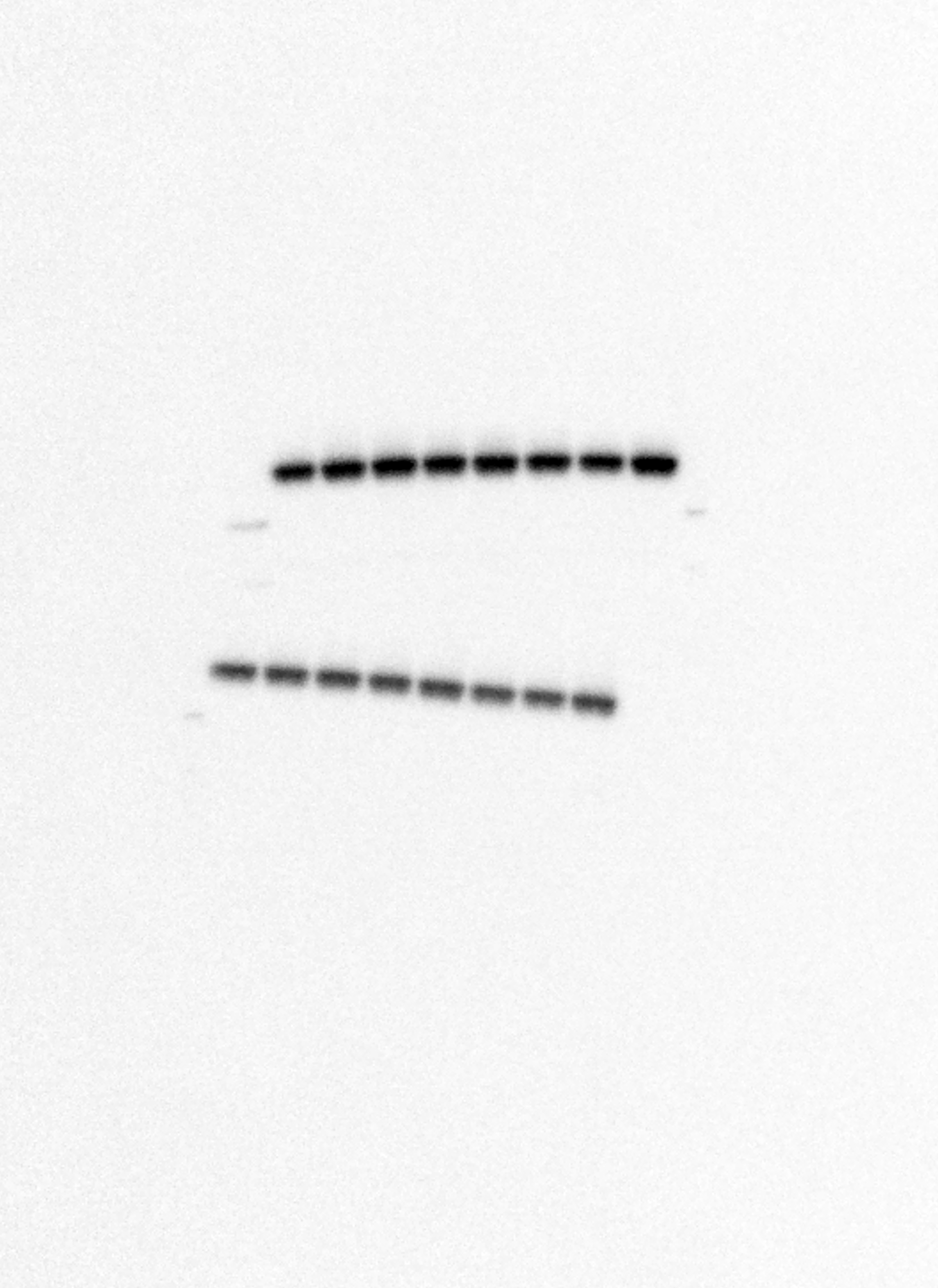

Supplement: Supplementary file 9 — Appendix + EV source data [file 44318_2025_498_MOESM9_ESM.zip › SD_EV_Appendix/EMBOJ-2024-119900_FigEV4_Westernblot_files/GluN2A/Blot1_tubulinforGluN2A.tif]

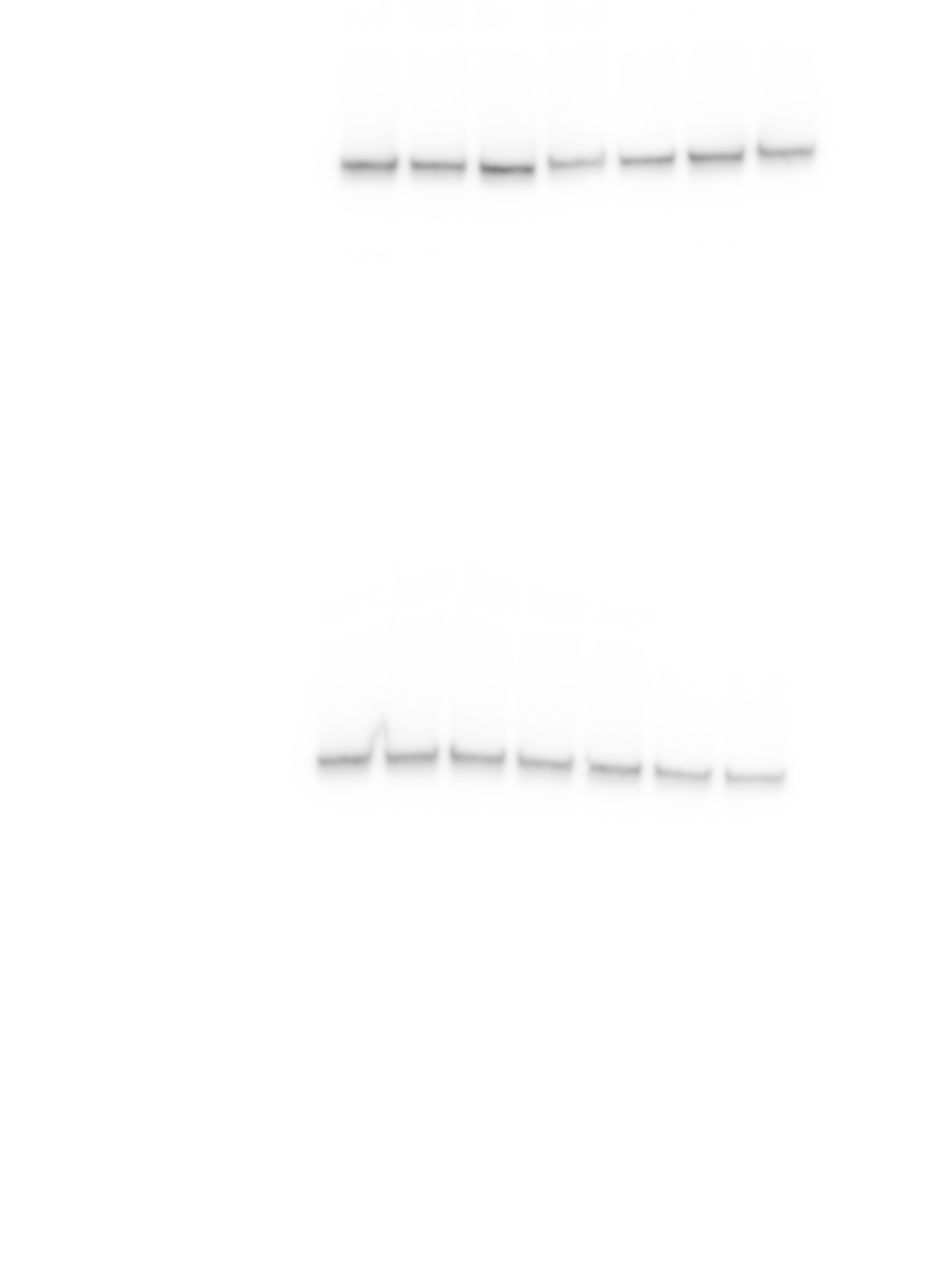

Supplement: Supplementary file 9 — Appendix + EV source data [file 44318_2025_498_MOESM9_ESM.zip › SD_EV_Appendix/EMBOJ-2024-119900_FigEV4_Westernblot_files/GluN2A/Blot2-2'_GluN2A.tif]

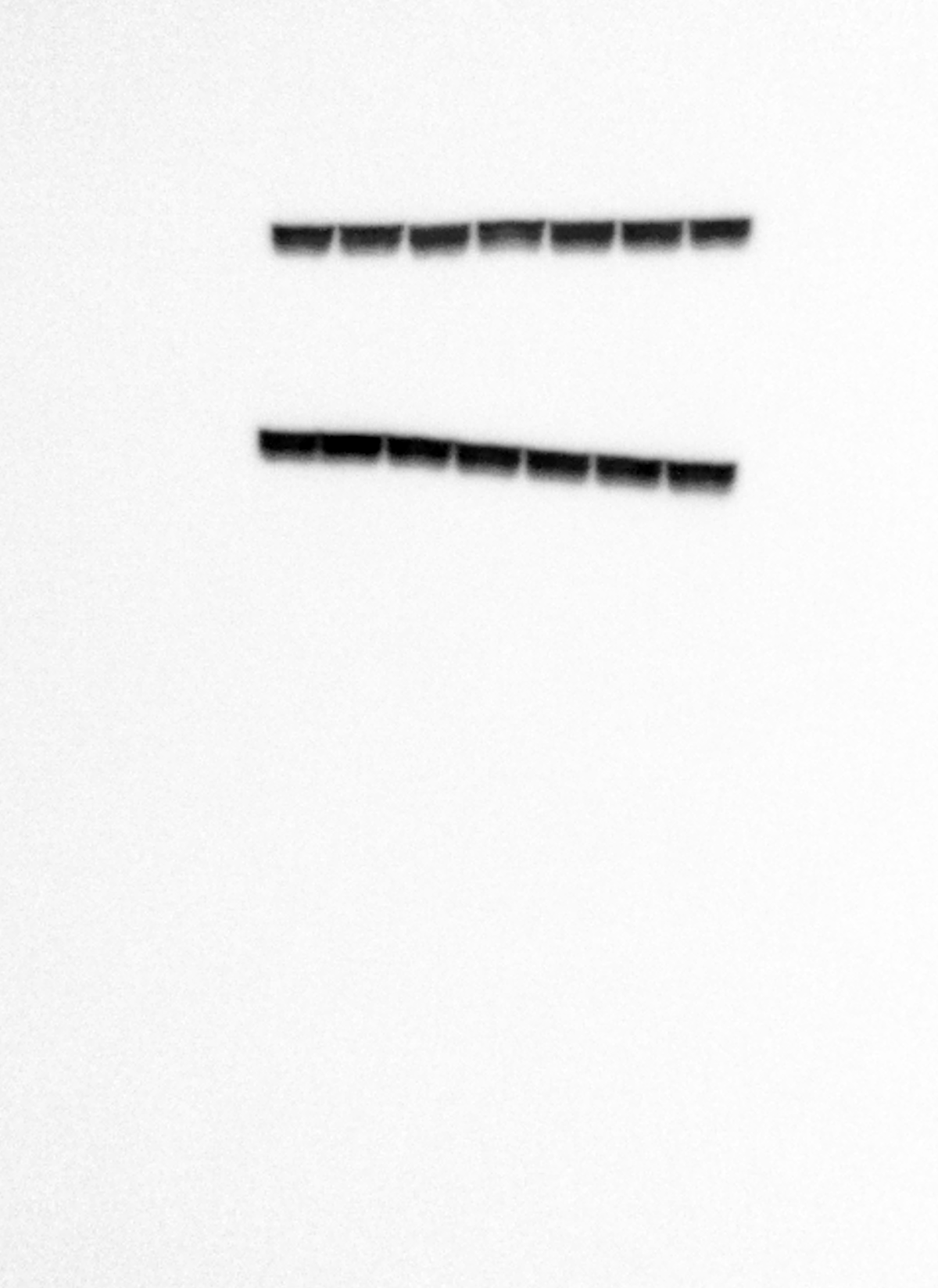

Supplement: Supplementary file 9 — Appendix + EV source data [file 44318_2025_498_MOESM9_ESM.zip › SD_EV_Appendix/EMBOJ-2024-119900_FigEV4_Westernblot_files/GluN2A/Blot2-2'_tubulinforGluN2A.tif]

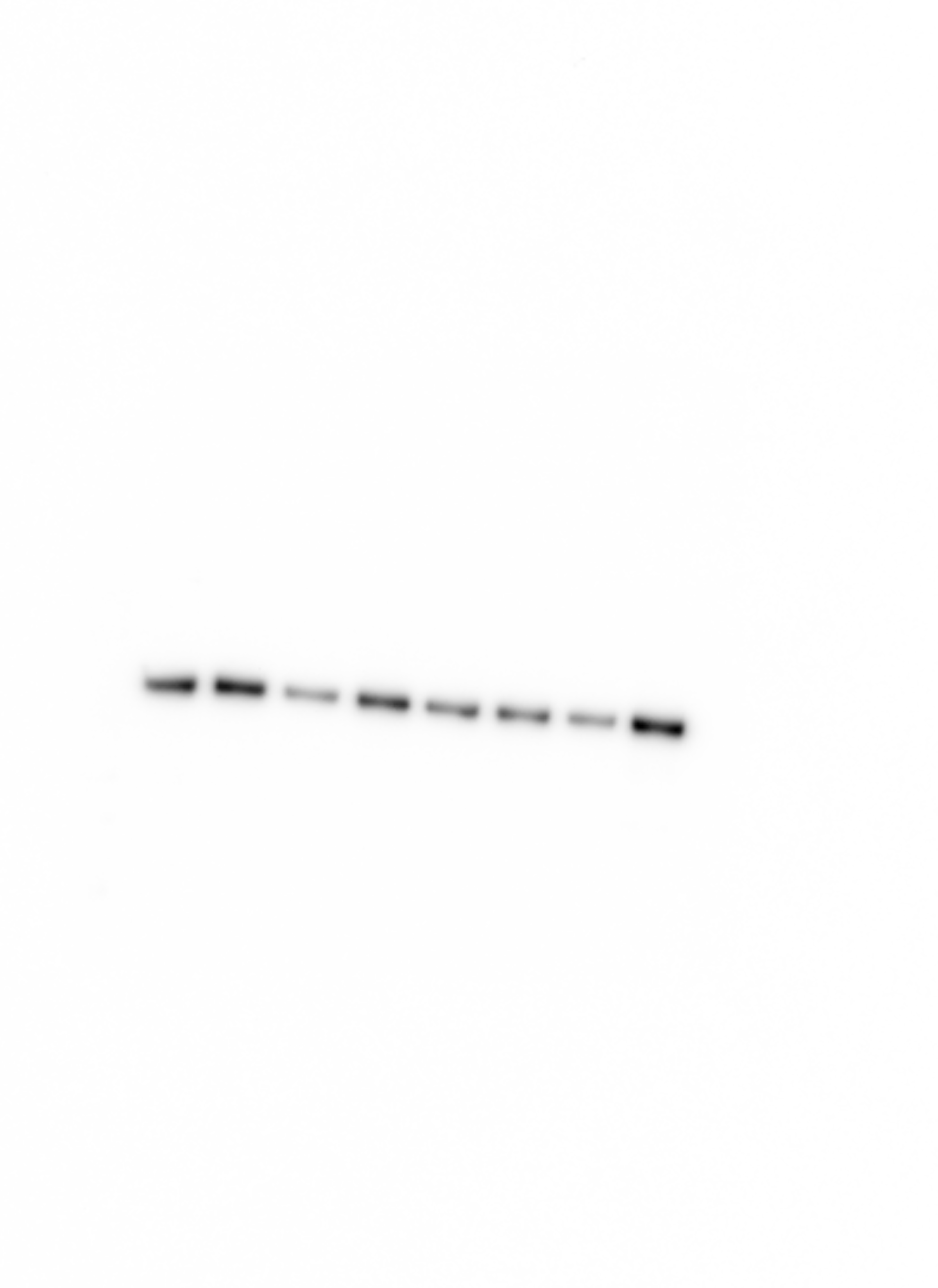

Supplement: Supplementary file 9 — Appendix + EV source data [file 44318_2025_498_MOESM9_ESM.zip › SD_EV_Appendix/EMBOJ-2024-119900_FigEV4_Westernblot_files/GluN2B/Blot1_GluN2B.tif]

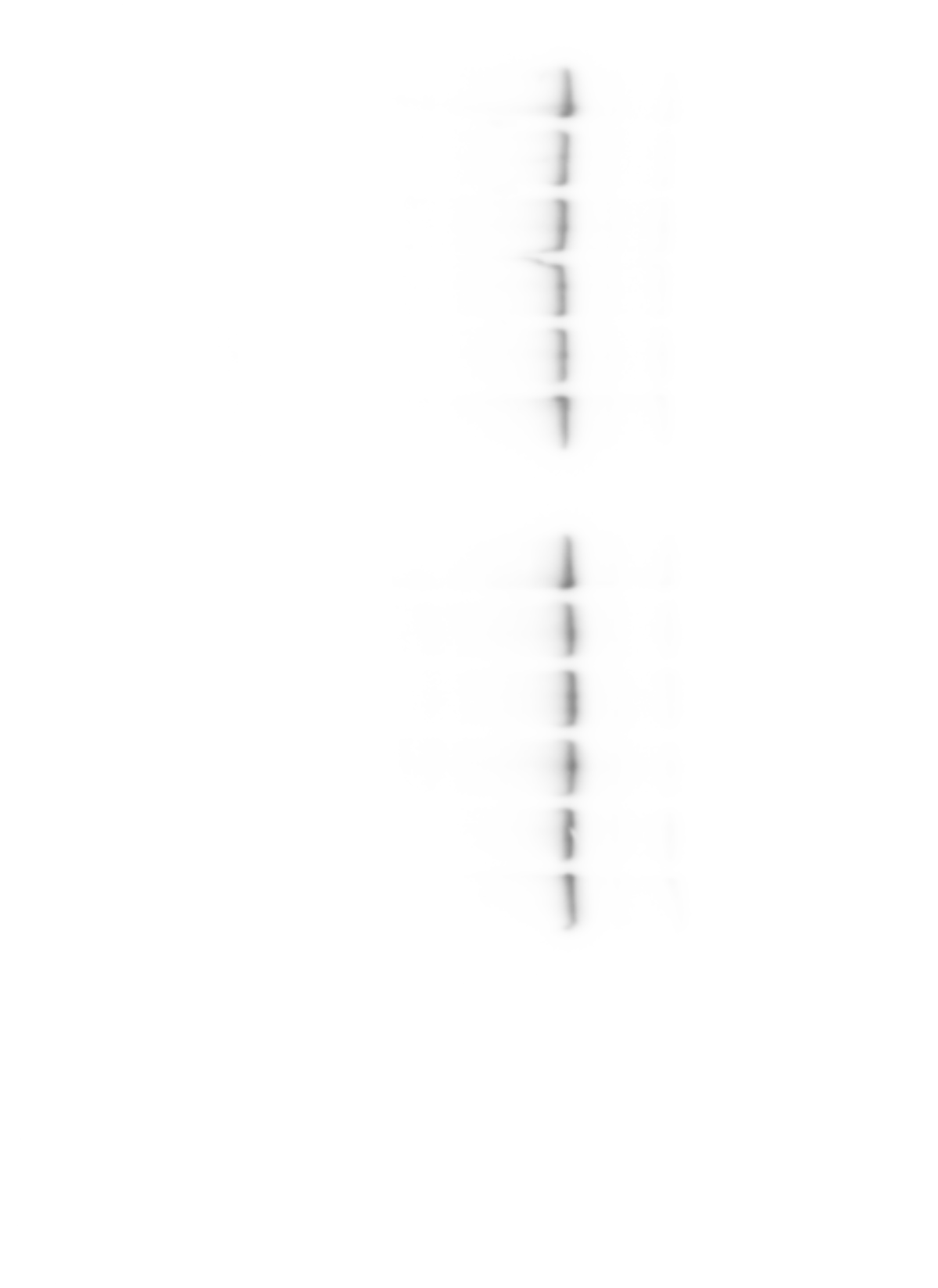

Supplement: Supplementary file 9 — Appendix + EV source data [file 44318_2025_498_MOESM9_ESM.zip › SD_EV_Appendix/EMBOJ-2024-119900_FigEV4_Westernblot_files/GluN2B/Blot2-2'_GluN2B.tif]

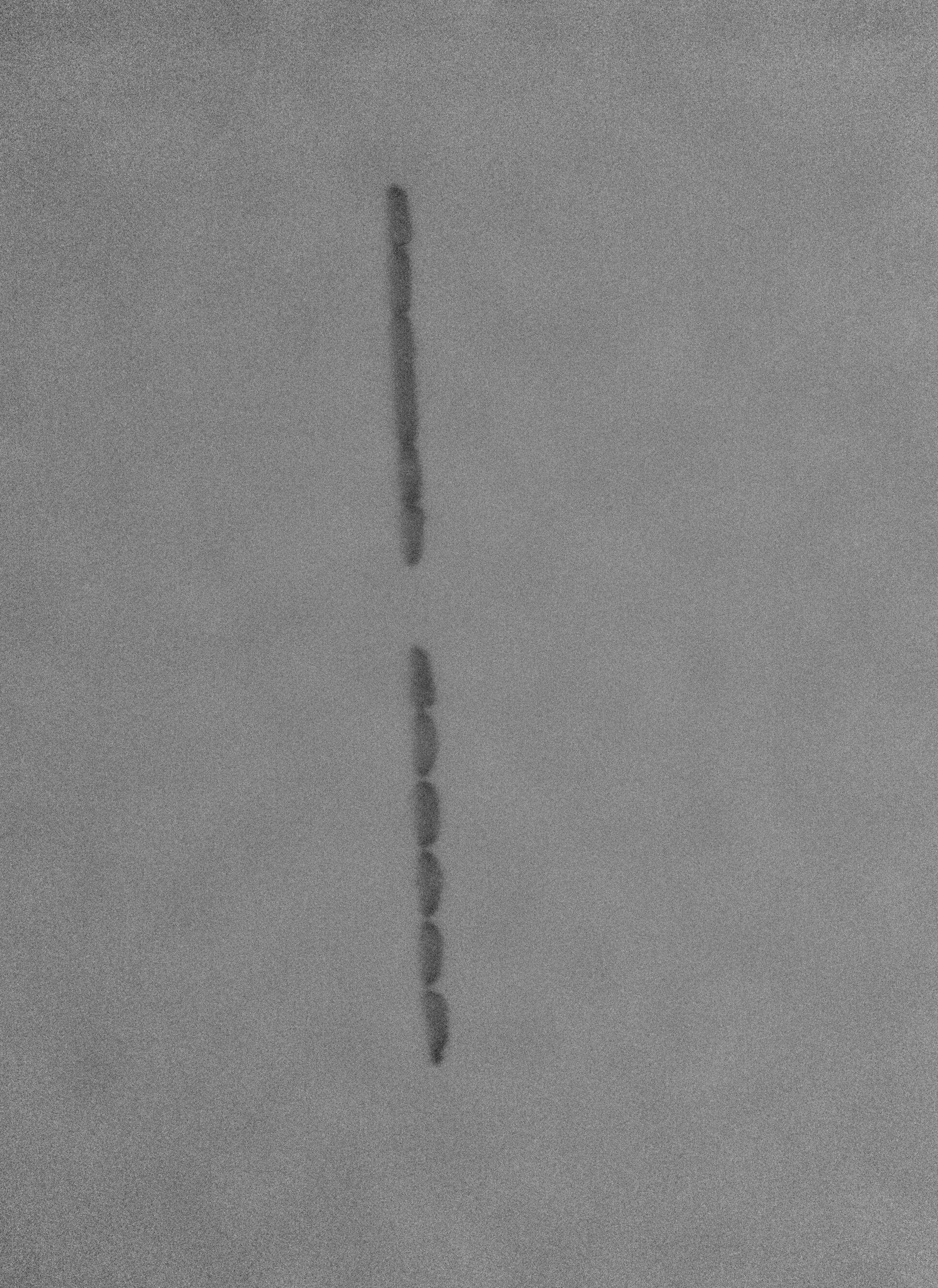

Supplement: Supplementary file 9 — Appendix + EV source data [file 44318_2025_498_MOESM9_ESM.zip › SD_EV_Appendix/EMBOJ-2024-119900_FigEV4_Westernblot_files/GluN2B/Blot2-2'_tubulinforGluN2B.tif]
