# Supplementary material for: Optochemical profiling of NMDAR molecular diversity at synaptic and extrasynaptic sites
Source: EMBO J. 2025 Jul 8;44(16):4577–610. doi: 10.1038/s44318-025-00498-x (PMC12361563; doi:10.1038/s44318-025-00498-x)
Supplement: Supplementary file 10 — Expanded View Figures [file 44318_2025_498_MOESM10_ESM.pdf]

Expanded View Figures

Figure EV1. (related to Fig. 1): Photochemical properties of MASp and design of the labeling conditions in Xenopus oocytes.

(**A**) UV-visible spectra of MASp conjugated to L-cysteine ($MASp_{Cys}$, see "Methods" and Appendix Fig. S6) in oocyte recording medium (pH 7.3, 33 µM MASp) in the dark (black trace) and under illumination with increasing wavelengths, from 365 nm (dark violet) to 635 nm (red). Inset, absorbance at 358 nm (*trans*-$MASp_{Cys}$ absorbance peak) as a function of the illumination wavelength. (**B**) Thermal stability of *cis*-$MASp_{Cys}$. After recording a UV-visible spectrum of $MASp_{Cys}$ in the dark (mainly *trans* state, black trace), $MASp_{Cys}$ was irradiated for 10 min by 365 nm light to yield mainly *cis*-$MASp_{Cys}$ (violet spectrum). Spectra in violet/grey gradation represent the gradual *cis*-to-*trans* transition of $MASp_{Cys}$ in the dark at different time points (up to ~8 h) post UV irradiation. Inset, evolution over time of the absorbance at 358 nm. Single-exponential fit (grey line) yielded a time constant $\tau = 259$ min (~4 h) for *cis*-to-*trans* $MASp_{Cys}$ thermal relaxation in the dark. (**C**) HPLC chromatograms monitored at the isosbestic point (440 nm) of the photostationary states (PSS) of $MASp_{Cys}$ in the dark (black trace), after illumination with 365 nm light (violet trace) and subsequent illumination with 525 nm light (green trace). (**D**) Experimental workflow for heterologous expression of GluN1/GluN2B receptors, MASp labeling and photomodulation of NMDAR activity in Xenopus oocytes. (**E–H**) MASp binds to the endogenous cysteine GluN2B-C395. (**E**) Left, schematic of a GluN1/GluN2B dimer with the positions of the free cysteines (i.e. not involved in disulfide bridges) highlighted in yellow. Right, inhibition traces by MK-801 (100 nM), an open channel pore blocker, of unlabeled (− MASp, black) and labeled (+ MASp, grey) GluN1/GluN2B WT NMDARs kept in the dark (MASp in its *trans* state). (**F**) Superposition of the normalized MK801 inhibition traces from (**E**). Note the increase of MK-801 inhibition rate after labeling with MASp, indicating an increase in the open probability when GluN1/GluN2B NMDARs are conjugated with MASp. (**G**) Left, neutralization of the reactivity of C395, a cysteine located in GluN2B NTD-ABD linker, by mutation into a serine. Right, superposed and normalized MK-801 inhibition traces for labeled (+ MASp, grey) and unlabeled (- MASp, black) GluN1/GluN2B-C395S mutants. Note that on this mutant, labeling does not affect the rate of inhibition by MK-801. (**H**) Summary of the rates of inhibition by MK-801. GluN1/GluN2B WT: -MASP, $n = 5$ cells; +MASP, $n = 6$ cells. GluN1/GluN2B-C395S: -MASP, $n = 6$ cells; +MASP, $n = 5$ cells. Data are displayed as mean ± s.e.m. n.s., $P > 0.05$; *$P < 0.05$; multiple Mann–Whitney tests, P values were adjusted for multiple comparisons using Bonferroni correction. Only the pre-selected indicated comparisons were performed. Exact P values are displayed in Dataset EV1. Source data are available online for this figure.

© The Author(s)

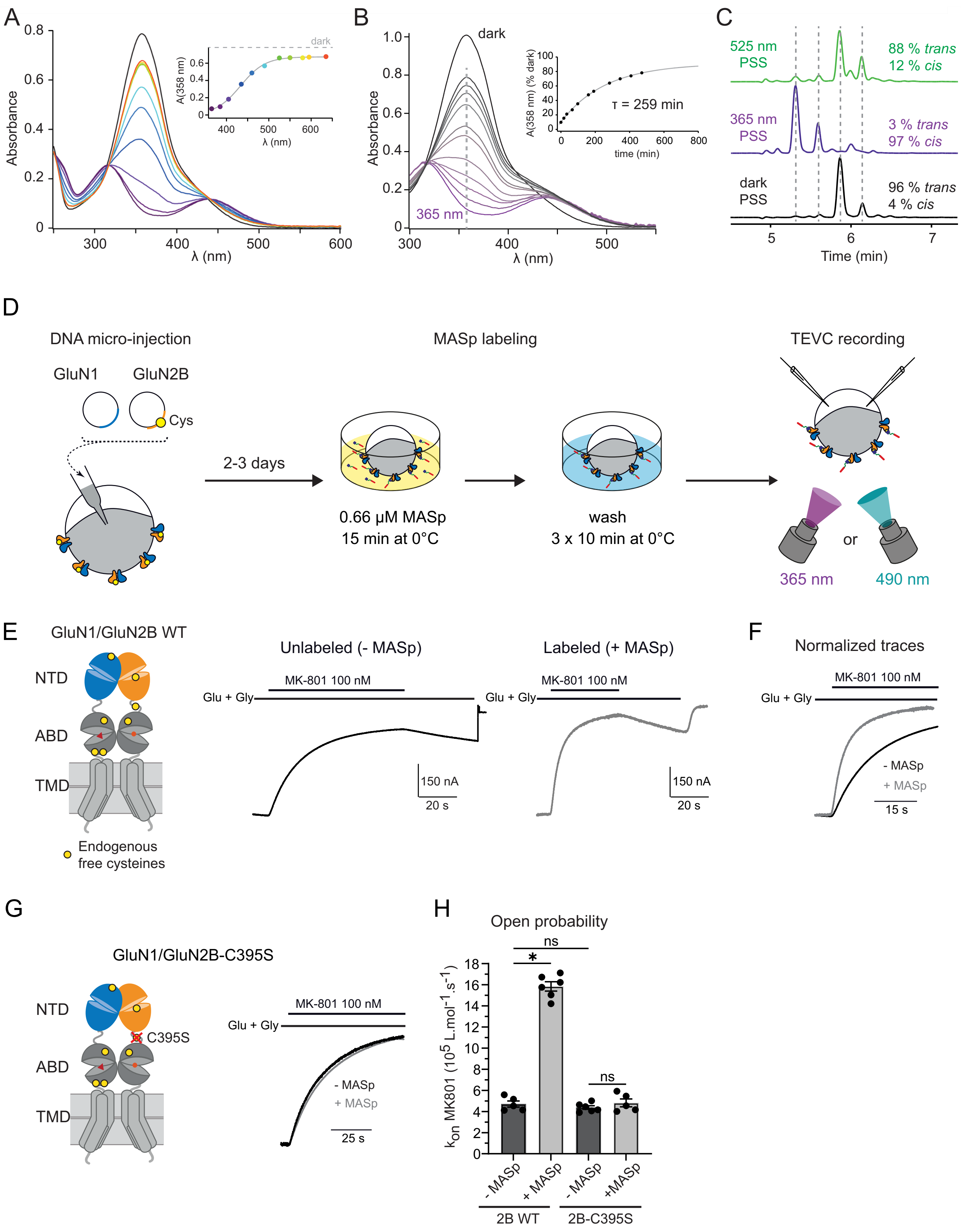
A
Absorbance
λ (nm)
A(358 nm)
dark
B
dark
365 nm
Absorbance
λ (nm)
A(358 nm) (% dark)
time (min)
τ = 259 min
C
525 nm PSS
88 % trans 12 % cis
365 nm PSS
3 % trans 97 % cis
dark PSS
96 % trans 4 % cis
Time (min)
D
DNA micro-injection
GluN1
GluN2B
Cys
2-3 days
MASp labeling
0.66 µM MASp 15 min at 0°C
wash 3 x 10 min at 0°C
TEVC recording
or
365 nm
490 nm
E
GluN1/GluN2B WT
NTD
ABD
TMD
Endogenous free cysteines
Unlabeled (- MASp)
Labeled (+ MASp)
MK-801 100 nM
Glu + Gly
150 nA
20 s
F
Normalized traces
- MASp
+ MASp
15 s
G
GluN1/GluN2B-C395S
C395S
25 s
H
Open probability
ns
*
k_{on} MK801 (10^5 L.mol^{-1}.s^{-1})
- MASp
+ MASp
2B WT
2B-C395S

 © The Author(s)

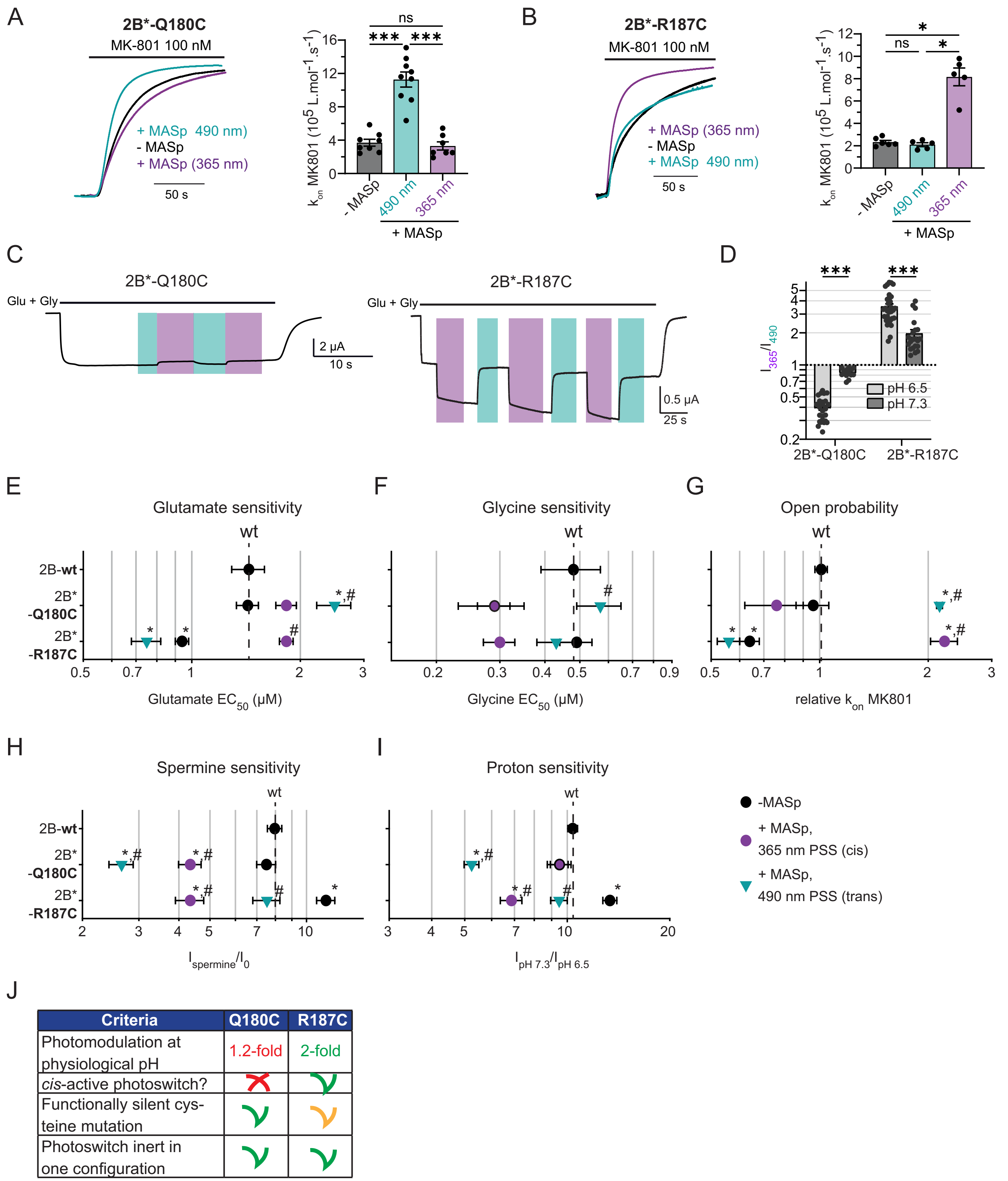
A
2B*-Q180C
MK-801 100 nM
+ MASp 490 nm)
- MASp
+ MASp (365 nm)
50 s
ns
*** ***
kon MK801 (10^5 L.mol^-1.s^-1)
- MASp
490 nm
365 nm
+ MASp
B
2B*-R187C
MK-801 100 nM
+ MASp (365 nm)
- MASp
+ MASp 490 nm)
50 s
*
ns *
C
2B*-Q180C
Glu + Gly
2 µA
10 s
2B*-R187C
Glu + Gly
0.5 µA
25 s
D
*** ***
I365/I490
pH 6.5
pH 7.3
2B*-Q180C 2B*-R187C
E
Glutamate sensitivity
wt
2B-wt
2B* -Q180C
2B* -R187C
Glutamate EC50 (µM)
F
Glycine sensitivity
wt
Glycine EC50 (µM)
G
Open probability
wt
relative kon MK801
H
Spermine sensitivity
wt
Ispermine/I0
I
Proton sensitivity
wt
IpH 7.3/IpH 6.5
-MASp
+ MASp, 365 nm PSS (cis)
+ MASp, 490 nm PSS (trans)
J
Criteria
Q180C
R187C
Photomodulation at physiological pH
1.2-fold
2-fold
cis-active photoswitch?
Functionally silent cysteine mutation
Photoswitch inert in one configuration

Figure EV2. (related to Fig. 1): Photomodulation and pharmacological properties of GluN2B*-Q180C and -R187C mutants.

(**A**, **B**) Evaluation of the relative Po of MASp-labeled GluN1/GluN2B*-Q180C (**A**) and GluN1/GluN2B*-R187C (**B**) under different light conditions. Left, superposed current traces following application of agonists and 100 nM MK-801 of unlabeled receptors (- MASp, black) and MASp-labeled receptors (+MASp) under 365 (violet, mostly *cis*-MASP) or 490 nm light (blue-green, mostly *trans*-MASp). Right, summary of MK-801 inhibition rates. Kinetics of MK801 inhibition (mean ± s.e.m., number of cells): 2B*-Q180C, $k_{on} = 3.7 \pm 0.4\ 10^5$ $L.mol^{-1}.s^{-1}$, *n* = 8 (- MASp); $11.3 \pm 0.9\ 10^5$ $L.mol^{-1}.s^{-1}$, *n* = 9 (+MASp, 490 nm), and $3.3 \pm 0.5\ 10^5$ $L.mol^{-1}.s^{-1}$, *n* = 7 (+MASp, 365 nm); 2B*-R187C, $k_{on} = 2.3 \pm 0.1\ 10^5$ $L.mol^{-1}.s^{-1}$, *n* = 6 (- MASp); $2.1 \pm 0.2\ 10^5$ $L.mol^{-1}.s^{-1}$, *n* = 5 (+MASp, 490 nm), and $8.2 \pm 1.6\ 10^5$ $L.mol^{-1}.s^{-1}$, *n* = 5 (+MASp, 365 nm). n.s., $P > 0.05$; *$P < 0.05$; ***$P < 0.001$; multiple Mann–Whitney tests, *P* values were adjusted for multiple comparisons using Bonferroni correction. When MASp was conjugated to GluN2B-Q180C in its *trans* configuration (under 490 nm light), channel Po increased by ~2-fold compared to unlabeled receptors, while labeling with *cis*-MASp (under UV light) produced no significant change in receptor activity. MASp thus acts as a *trans*-on photoswitch at position Q180C. In contrast, MASp conjugated to GluN2B-R187C had no effect on receptor channel Po under 490 nm light, while it increased Po by ~3.5-fold under UV-light. MASp thus acts as a *cis*-on photoswitch at position R187C (see Fig. 1E, right). (**C**, **D**) Photomodulation of MASp-labeled, GluN1/GluN2B*-Q180C and -R187C receptors at physiological pH. (**C**) Current traces from MASp-labeled GluN1/GluN2B*-Q180C (left) and GluN1/GluN2B*-R187C (right) at pH 7.3. (**D**) Summary of photomodulation ratios for the Q180C and R187C mutants at pH 6.5 and 7.3. Photomodulation values at pH 6.5 are from Fig. 1. ***$P < 0.001$ multiple Mann–Whitney tests, *P* values were adjusted for multiple comparisons using Bonferroni correction. Only the pre-selected indicated comparisons were performed. Photomodulation values (mean ± s.e.m.) and number of cells are summarized in Appendix Table S1. (**E–I**) Determination of the relative open probability and sensitivity to different pharmacological agents of unlabeled (- MASp) and labeled (+MASp) GluN1/GluN2B*-Q180C and GluN1/GluN2B*-R187C receptors compared to unlabeled WT GluN1/GluN2B receptors. *$P < 0.05$ between the condition and unlabeled WT GluN1/GluN2B receptors; #$P < 0.05$, between MASp-labeled mutant receptors under 365 or 490 nm light and unlabeled mutant receptors. One-way ANOVA followed by Tukey's test. Values (mean ± s.e.m.) are summarized in Appendix Table S2. (**J**) Pros and cons of photo-enhancing GluN2B-NMDARs via the Q180C or the R187C labeling position. The R187C is the most favored labeling position due to its strong photomodulation at physiological pH and the fact that MASp at this position is active in *cis*. Data are displayed as mean ± s.e.m. Exact *P* values are summarized in Dataset EV1. Source data are available online for this figure.

 © The Author(s)

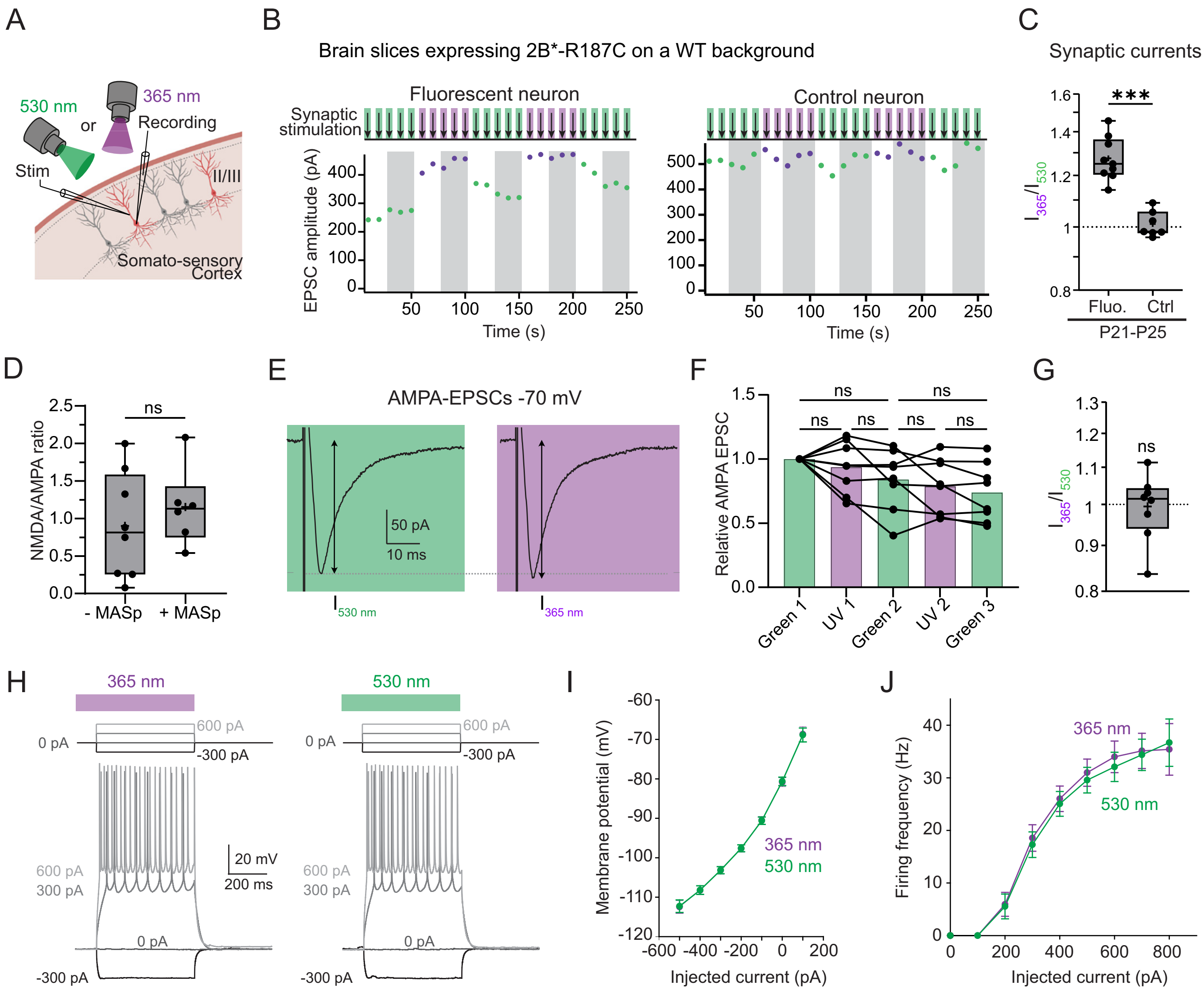

Figure EV3. (related to Fig. 3): Protocol of photomodulation of NMDA-EPSCs and determination of MASp background effects on brain slices.

(A) Data were obtained from electroporated (fluorescent) and non-electroporated (non-fluorescent, control) layer II/II pyramidal neurons from the somatosensory cortex upon stimulation at the level of their apical dendrite. **(B)** Top, protocol of photomodulation of NMDAR synaptic currents (NMDA-EPSCs). Synaptic stimulation was performed continuously at 0.1 Hz. 525 nm (green bars) or 365 nm (violet bars) illumination started 1 s before synaptic stimulation and ended 200 ms after synaptic stimulation (1.2 s total). Illumination cycles consisted of alternations between 5 stimulations under 525 nm light and 5 stimulations under 365 nm light. Bottom, amplitudes of NMDA-EPSCs from a MASp-labeled, fluorescent (expressing GluN2B*-R187C) neuron from a P14 mouse under alternating 525 nm (green points) and 365 nm (violet points) illumination, showing reversible and reproducible photomodulation of NMDA-EPSCs. Given the slow reversion of EPSC amplitude by 525 nm light, only the three last EPSCs of each 5-stimulation cycle were considered for amplitude quantification and trace averaging (grey bars). **(C)** Photomodulation ratios (I_{365}/I_{530}) of NMDA-EPSCs of MASp-labeled, fluorescent and control neurons from P21-P25 animals. Photomodulation values (mean ± s.e.m.) and number of cells are summarized in Appendix Table S6. **(D–J)** MASp labeling has no or minimal impact on synaptic transmission and neuronal electrical properties. **(D)** NMDA/AMPA ratios for WT neurons either unlabeled (- MASp, $n = 8$ cells) or labeled (+ MASp, $n = 6$ cells) from P21-P25 animals. n.s., $P > 0.05$, Mann–Whitney test. **(E–G)** No photodependent effect of MASp labeling on AMPA-EPSCs. **(E)** Representative AMPA-EPSCs (recorded at −70 mV) from a MASp-labeled control (non-fluorescent) layer II/III cortical neuron under 530 nm (green bar, left) or 365 nm light (violet bar, right) in a slice from a P21 mouse. Same illumination conditions as in **(B)**. **(F)** Summary of AMPA-EPSC amplitudes of MASp-labeled control neurons from P21-P22 mice under alternating 530 nm (green bars) and 365 nm (violet bars) illumination. Each dot represents the average amplitude of AMPA-EPSCs for each cycle for each cell. $n = 8$ cells. n.s., $P > 0.05$; Repeated measures Anova followed by Tukey's multiple comparison test. **(G)** Photomodulation ratios of AMPA-EPSCs from MASp-labeled, control neurons, showing no significant photomodulation. Quantification of the photomodulation ratio was performed similarly to Fig. 3. $n = 8$ cells. n.s., $P > 0.05$; one sample Wilcoxon test against the value of 1. **(H–J)** No photodependence of the excitability properties of MASp-labeled control neurons. **(H)** Current-clamp voltage traces of a MASp-labeled, control neuron from a P23 mouse during 500 ms current injection steps under UV (365 nm, violet bar) and green light (530 nm, green bar). UV light was applied 50 ms before and during the current step, while green light was applied 2 s before and during the current step. The amount of current injected is written next to the corresponding voltage trace. **(I, J)** No light-dependence of the membrane potential (**(I)**, $n = 7–8$ cells) and firing frequency (**(J)**, $n = 5–7$ cells) as a function of injected current after MASp labeling of control neurons from P21-P24 animals. All recordings in brain slices were performed at physiological pH. Data are displayed as mean ± s.e.m. Box plots: centerlines show the median; crosses show the mean; box limits indicate the 25th and 75th percentiles; whiskers extend to the minimum and maximum. Exact *P* values are summarized in Dataset EV1. Source data are available online for this figure.

© The Author(s)

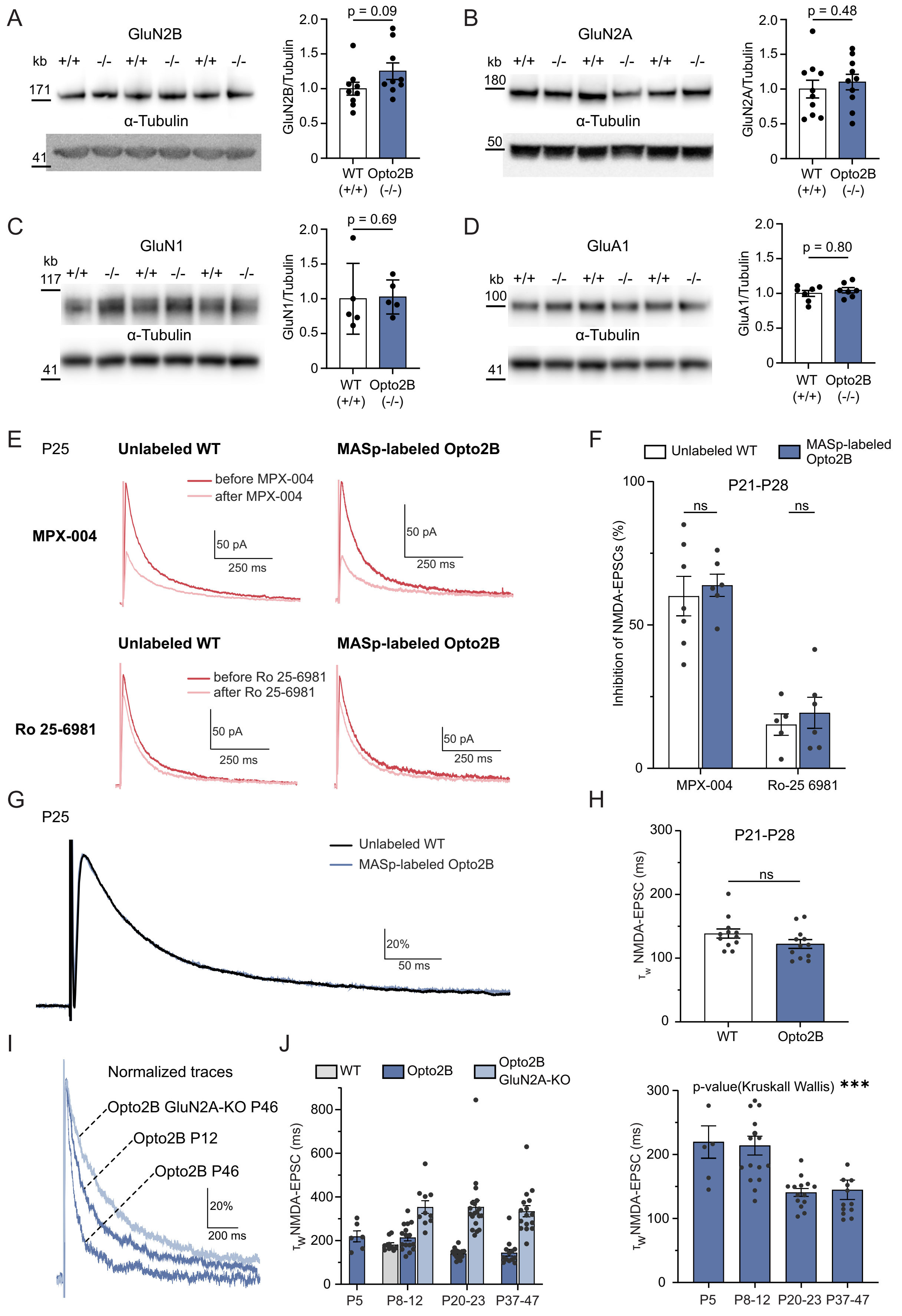
A
GluN2B
kb +/+ -/- +/+ -/- +/+ -/-
171
α-Tubulin
41
p = 0.09
GluN2B/Tubulin
WT (+/+) Opto2B (-/-)
B
GluN2A
180
α-Tubulin
50
p = 0.48
GluN2A/Tubulin
C
GluN1
117
41
p = 0.69
GluN1/Tubulin
D
GluA1
100
41
p = 0.80
GluA1/Tubulin
E
P25
Unlabeled WT
MASp-labeled Opto2B
before MPX-004
after MPX-004
MPX-004
50 pA
250 ms
before Ro 25-6981
after Ro 25-6981
Ro 25-6981
F
Unlabeled WT
MASp-labeled Opto2B
P21-P28
ns
Inhibition of NMDA-EPSCs (%)
MPX-004
Ro-25 6981
G
P25
Unlabeled WT
MASp-labeled Opto2B
20%
50 ms
H
P21-P28
ns
τw NMDA-EPSC (ms)
WT
Opto2B
I
Normalized traces
Opto2B GluN2A-KO P46
Opto2B P12
Opto2B P46
20%
200 ms
J
WT
Opto2B
Opto2B GluN2A-KO
τw NMDA-EPSC (ms)
P5
P8-12
P20-23
P37-47
p-value(Kruskall Wallis) ***

 © The Author(s)

Figure EV4. (related to Fig. 4): Similar NMDAR subunit profiles between Opto2B and WT mice.

(**A–D**) No significant difference of expression of NMDAR subunits GluN2B (**A**), GluN2A (**B**), and GluN1 (**C**), and of AMPAR subunit GluA1 (**D**) in P46-54 Opto2B mice (−/−) and their wt littermates (+/+). Quantification was performed by dividing the intensity of the subunit band (upper lane) by the intensity of the corresponding α-tubulin band (lower lane). Subunit/tubulin ratios were normalized to the average ratio of WT animals from the same blot. Uncropped blots are available in the Source Data files. $P > 0.05$ for all conditions, Mann–Whitney test. $n = 9$ WT and 9 Opto2B animals for GluN2B, 10 WT and 10 Opto2B animals for GluN2A, 5 WT and 5 Opto2B animals for GluN1, and 7 WT and 7 Opto2B animals for GluA1. (**E**) Top, superposition of NMDA-EPSCs of CA1 pyramidal neurons before and after application of MPX-004 (top) or Ro 25-6981 (bottom) from unlabeled WT (left) and MASp-labeled Opto2B mice (right) at age P25, recorded in the dark (MASp in its inactive, *trans* configuration). (**F**) Summary of NMDA-EPSC inhibition (%) by MPX-004 or Ro 25-6981 of CA1 pyramidal neurons from unlabeled WT (white) and MASp-labeled Opto2B mice (blue) at P21-P28. MPX-004: $n = 7$ cells for unlabeled WT and 6 cells for MASP-labeled Opto2B. Ro 25-6981: $n = 5$ cells for unlabeled WT and 6 for MASp-labeled Opto2B. n.s., $P > 0.05$; multiple Mann–Whitney tests, *P* values were adjusted using Bonferroni correction. Only the pre-selected indicated comparisons were performed. (**G**) Superposition of normalized NMDA-EPSCs of CA1 pyramidal neurons from unlabeled WT (black) and MASp-labeled Opto2B mice (blue) at age P25. (**H**) Summary of NMDA-EPSC decay kinetics from unlabeled WT (white) and MASp-labeled Opto2B mice (blue) at P21-P28. $N = 12$ cells for unlabeled WT and 6 cells for MASp-labeled Opto2B. n.s., $P > 0.05$; Mann–Whitney test. Note the similar NMDA-EPSC decay time between WT and Opto2B animals, suggesting that the R187C mutation does not perturb developmental maturation of NMDA subtypes. (**I**) Superposition of normalized NMDA EPSCs of MASp-labeled CA1 pyramidal neurons from Opto2B mice at age P12 and P46, as well as from P46 Opto2B/GluN2A KO animals, recorded under green light (MASp in its inactive configuration). Each trace is the average of 12 individual EPSCs. (**J**) Left, decay time constants (τ_w NMDA-EPSC) of NMDA-EPSCs of MASp-labeled CA1 pyramidal neurons from WT (grey), Opto2B (dark blue) and Opto2B GluN2A-KO (light blue) mice at different age ranges. Note the absence of significant difference between the NMDA-EPSC decay kinetics of from WT and Opto2B animals at P8-P12, suggesting similar NMDAR subunit populations. NMDA-EPSC decay kinetics from Opto2B/GluN2A KO mice remained slow at older ages, consistently with what was previously shown for animals lacking GluN2A expression (Gray et al, 2011). Right, zoom on the Opto2B mouse condition for better visualization. ***$P < 0.001$, Kruskall-Wallis test on Opto2B condition. Note the acceleration of NMDA-EPSC decay time with age on Opto2B mice, which is consistent with what was observed in the literature on WT animals (Paoletti et al, 2013). Cell numbers for each condition are indicated in Dataset EV1. All the recordings on brain slices were performed at physiological pH. All data are displayed as mean ± s.e.m. Exact *P* values are summarized in Dataset EV1. Source data are available online for this figure.

© The Author(s)

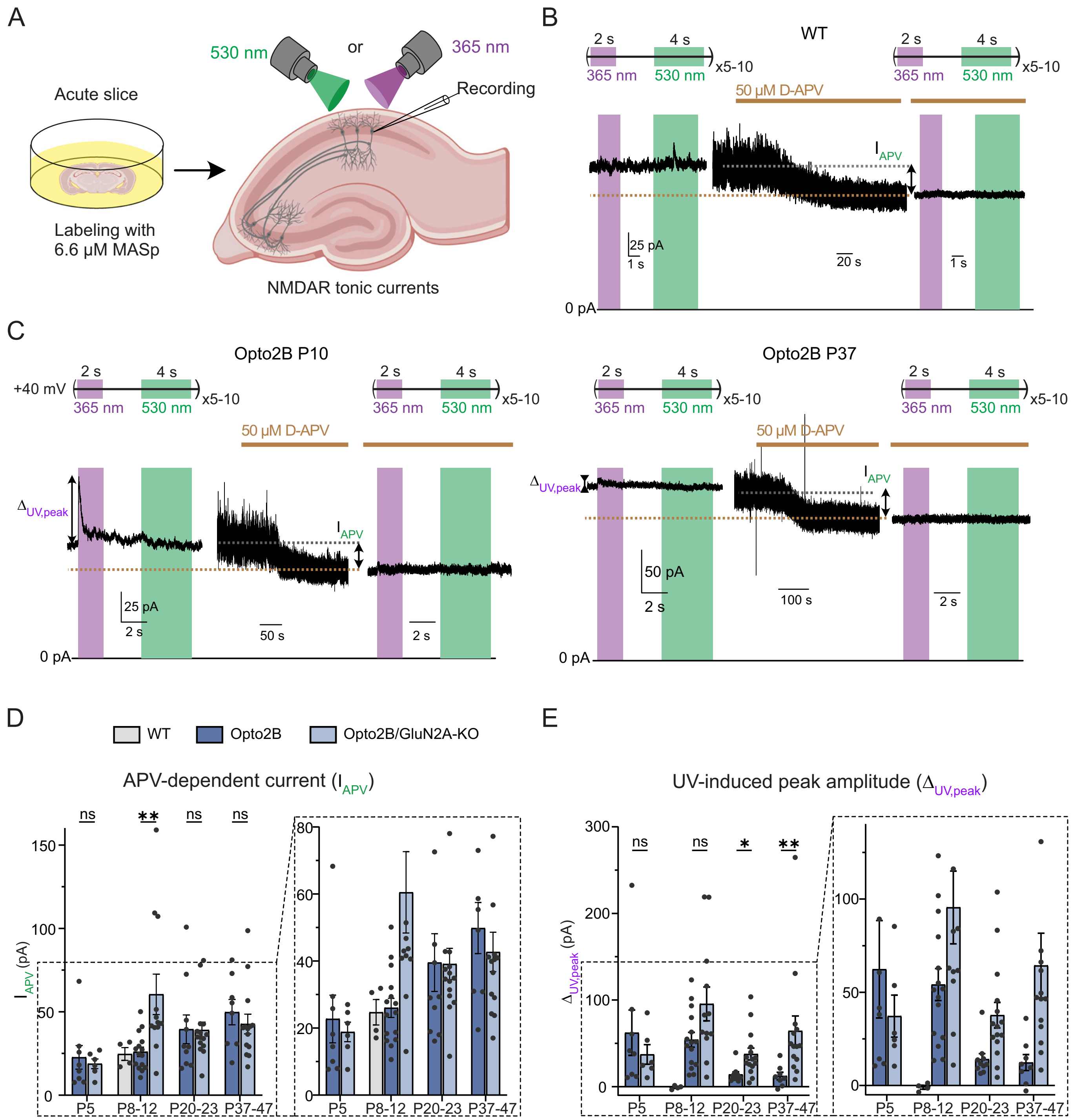

Figure EV5. (related to Fig. 5): Determination of the photomodulation ratio of NMDA tonic currents.

(**A**) Protocol of tonic current photomodulation. Note the absence of electrical stimulation: recorded tonic currents are mediated by low levels of tonic glutamate. See "Methods" and Main Text for more details. (**B**) Tonic current trace of a MASp-labeled, CA1 pyramidal neuron from a P10 WT mouse before (left) and during (middle and right) application of 50 µM D-APV. Left and right traces are the average of 5 to 10 traces. Note the absence of photomodulation. (**C**) Tonic current traces of MASp-labeled, CA1 pyramidal neurons from a P10 (left) or a P37 (right) Opto2B mouse before and during application of 50 µM D-APV. Left and right traces are the average of 5 to 10 traces. Displayed arrows indicate the currents measured in the following panels and used to calculate the photomodulation ratio (see also Fig. 3). (**D**) Amplitude of APV-sensitive currents (as shown in (**A**)) of MASp-labeled, CA1 pyramidal neurons from WT (grey), Opto2B (dark blue) and Opto2B GluN2A-KO (light blue) mice according to age ranges. Right, zoom on data for better visibility of bar graphs. (**E**) Amplitude of the UV-induced peak (as shown in (**A**)) of MASp-labeled, CA1 pyramidal neurons from WT (grey), Opto2B (dark blue) and Opto2B GluN2A-KO (light blue) mice according to age ranges. Right, zoom on data for better visibility of bar graphs. All recordings in brain slices were performed at physiological pH. Data are displayed as mean ± s.e.m. Cell numbers for each condition and exact *P* values are summarized in Dataset EV1. Source data are available online for this figure.

 © The Author(s)
